# Triage in major incidents: development and external validation of novel machine learning-derived primary and secondary triage tools

**DOI:** 10.1136/emermed-2022-212440

**Published:** 2023-09-26

**Authors:** Yuanwei Xu, Nabeela Malik, Saisakul Chernbumroong, James Vassallo, Damian Keene, Mark Foster, Janet Lord, Antonio Belli, Timothy Hodgetts, Douglas Bowley, George Gkoutos

**Affiliations:** 1 Centre for Computational Biology, Institute of Cancer and Genomic Sciences, University of Birmingham, Birmingham, UK; 2 Health Data Science Centre, University of Birmingham, Birmingham B15 2TT, UK; 3 NIHR Surgical Reconstruction Microbiology Research Centre, Edgbaston, UK; 4 University Hospitals Birmingham NHS Foundation Trust, Birmingham, UK; 5 Institute of Inflammation and Ageing, University of Birmingham, Birmingham, UK; 6 Academic Department of Military Surgery & Trauma, Royal Centre for Defence Medicine, Mindelsohn Way, Edgbaston, Birmingham B152WB, UK; 7 Emergency Department, Derriford Hospital, Plymouth, UK; 8 Academic Department of Military Emergency Medicine, Royal Centre for Defence Medicine, Mindelsohn Way, Edgbaston, Birmingham B15 2WB, UK; 9 UK Strategic Command, Northwood Headquarters, Northwood, UK; 10 Institute of Translational Medicine, University Hospitals Birmingham NHS Foundation Trust, Birmingham, UK; 11 MRC Health Data Research UK (HDR UK), Birmingham, UK

**Keywords:** major incident, disaster planning, major trauma management, triage, pre-hospital care

## Abstract

**Background:**

Major incidents (MIs) are an important cause of death and disability. Triage tools are crucial to identifying priority 1 (P1) patients—those needing time-critical, life-saving interventions. Existing expert opinion-derived tools have limited evidence supporting their use. This study employs machine learning (ML) to develop and validate models for novel primary and secondary triage tools.

**Methods:**

Adults (16+ years) from the UK Trauma Audit and Research Network (TARN) registry (January 2008–December 2017) served as surrogates for MI victims, with P1 patients identified using predefined criteria. The TARN database was split chronologically into model training and testing (70:30) datasets. Input variables included physiological parameters, age, mechanism and anatomical location of injury. Random forest, extreme gradient boosted tree, logistic regression and decision tree models were trained to predict P1 status, and compared with existing tools (Battlefield Casualty Drills (BCD) Triage Sieve, CareFlight, Modified Physiological Triage Tool, MPTT-24, MSTART, National Ambulance Resilience Unit Triage Sieve and RAMP). Primary and secondary candidate models were selected; the latter was externally validated on patients from the UK military’s Joint Theatre Trauma Registry (JTTR).

**Results:**

Models were internally tested in 57 979 TARN patients. The best existing tool was the BCD Triage Sieve (sensitivity 68.2%, area under the receiver operating curve (AUC) 0.688). Inability to breathe spontaneously, presence of chest injury and mental status were most predictive of P1 status. A decision tree model including these three variables exhibited the best test characteristics (sensitivity 73.0%, AUC 0.782), forming the candidate primary tool. The proposed secondary tool (sensitivity 77.9%, AUC 0.817), applicable via a portable device, includes a fourth variable (injury mechanism). This performed favourably on external validation (sensitivity of 97.6%, AUC 0.778) in 5956 JTTR patients.

**Conclusion:**

Novel triage tools developed using ML outperform existing tools in a nationally representative trauma population. The proposed primary tool requires external validation prior to consideration for practical use. The secondary tool demonstrates good external validity and may be used to support decision-making by healthcare workers responding to MIs.

**WHAT IS ALREADY KNOWN ON THIS TOPIC**

- During major incidents (MIs) (eg, terrorist attacks), triage tools have a crucial role in maximising overall survival by identifying priority 1 (P1) patients.
- Existing tools, derived using expert opinion, have limited evidence to support their use.

**WHAT THIS STUDY ADDS**

- In this study, novel machine learning-based primary and secondary triage tools surpassed the current UK National Ambulance Resilience Unit Triage Sieve and other existing tools in identifying P1 patients within a nationally representative trauma population.
- The secondary tool demonstrated favourable external validity. However, the primary tool could not be externally validated due to missing GCS component data.

**HOW THIS STUDY MIGHT AFFECT RESEARCH, PRACTICE OR POLICY**

- The proposed secondary tool, applicable using a portable device, may be used to support decision-making among healthcare workers responding to MIs.

## Introduction

In the immediate aftermath of a major incident (MI), patient needs exceed the resources available to treat them^1–5^: triage tools seek to categorise patients, to guide the order of treatment, transport from the scene and the choice of medical facility for definitive care.^5 6^ A vital function of triage tools is to identify patients requiring time-critical, life-saving interventions (priority 1 or P1 patients). Failure to identify these patients (undertriage) is associated with absolute harm arising from delays in care or selection of an inappropriate medical facility.^6 7^ However, overtriage may risk overwhelming healthcare facilities with patients not requiring time-critical treatment.^2^

Primary triage, conducted at the scene of an MI, uses paper-based flow diagrams that are quick and simple to apply under challenging conditions.^8^ Existing primary triage tools have largely been developed using expert opinion, often with limited evidence to support their use.^6^ These include the National Ambulance Resilience Unit (NARU) Triage Sieve (current UK tool for adults), the Australian CareFlight and the US Simple Triage And Rapid Treatment (START).^6 9 10^ These tools use ambulatory status to designate priority 3 (minor) category, followed by physiological assessments to distinguish P1 from P2 (less critical) patients. A recent study demonstrated that the UK military’s Battlefield Casualty Drills (BCD) Triage Sieve attained greatest sensitivity among 10 international primary triage tools in detecting P1 status among adults; however, this was associated with an overtriage rate of 72%.^10^

Primary triage is often, but not always, followed by a further targeted prehospital clinical assessment of patients known as secondary triage. This is usually undertaken in a place of relative safety (eg, Casualty Clearing Station or hospital reception area)^1 8^; thus, the additional use of medical equipment and/or portable devices is more plausible. Two existing secondary MI triage tools are the UK’s Major Incident Medical Management and Support Triage Sort which has suboptimal sensitivity (15.7%) in predicting the need for life-saving intervention,^11^ and the US points-based Sacco Triage Method (developed to predict mortality) which is time-consuming and complex to apply.^9^

Anatomical assessment of injuries has yet to feature in any MI triage tool, yet this is commonly used in the field triage of singly injured patients.^12^ Advanced age is associated with worse outcomes following injury; however, existing tools do not incorporate this in patient assessment.^13^ There is scope to develop evidence-based primary and secondary MI triage tools which offer greater sensitivity while decreasing overtriage compared with the BCD Triage Sieve, yet preserve applicability. Tree-based machine learning models have demonstrated utility in clinical risk stratification, with the ability to capture non-linear interactions between input variables.^14 15^ This study aimed to develop machine learning models that can be adapted into primary and secondary MI triage tools and to externally validate these models using an independent population of injured patients.

## Methods

### Database for model training and internal testing

Model development and validation were conducted according to Transparent Reporting of a multivariable prediction model for Individual Prognosis or Diagnosis guidelines.^16^ Adult (16+ years) patients from the Trauma Audit and Research Network (TARN) registry presenting between 1 January 2008 and 31 December 2017 were included.^17^ The TARN registry prospectively captures prehospital and hospital data from 169 hospitals in England and Wales for patients who meet the following inclusion criteria: length of stay >72 hours or admission to intensive care and/or death in hospital.^17^ TARN does not include prehospital deaths. Patients for whom any input variables required for modelling were missing were excluded. Using hospital arrival dates recorded by TARN, the database was split temporally (70:30) to generate model training and internal testing datasets, respectively.

### Primary outcome of interest

The primary outcome of interest was P1 status, defined as the need for time-critical life or limb-saving surgery and/or advanced resuscitative measures.^18^ Each patient was retrospectively designated a triage category (priority 1, priority 2, priority 4/expectant or dead) (see flow diagram in online supplemental figure 1) using validated, consensus-derived definitions (table 1).^10 18^ Prior to the modelling phase, patients were designated either P1 or non-P1. The small numbers of P4 and dead patients (who share physiological similarities to P1 patients) were excluded from the modelling as these may impede model performance.

10.1136/emermed-2022-212440.supp1 Supplementary data

**Table 1:** Triage category definitions

Dead	Cardiac and/or respiratory arrest at initial prehospital evaluation that is not responsive to needle decompression or airway positioning (or the delivery of two rescue breaths in children less than 12 years old) Lack of palpable pulse and need for CPR (ie, cardiac arrest) within the first 15 min of EMS arrival on scene
Priority 4 (expectant)	In patients aged 0–49 years: third-degree (full thickness) burns to >90% of the body In patients aged 50 years and over: third-degree (full thickness) burns to >80% of the body Penetrating trauma to the head that crosses the midline with agonal respirations and/or no motor response, decorticate posturing or decerebrate posturing (ie, GCS Motor ≤3) Blunt trauma to the head with agonal respirations and/or no motor response, decorticate posturing or decerebrate posturing (ie, GCS Motor ≤3) Uncontrolled haemorrhage that resulted in cardiac arrest (defined as a lack of palpable pulse and EMS initiation of CPR) prior to EMS transport
Priority 1	Neurological, vascular or haemorrhage-controlling surgery to the head, neck or torso performed within 4 hours of arrival to hospital Limb-conserving surgery performed within 4 hours of arrival at hospital on a limb that was found to be pulseless distal to the injury prior to surgery Escharotomy performed on a patient with burns within 2 hours of arrival at a hospital Chest tube placed within 2 hours of arrival at hospital An advanced airway intervention (eg, intubation, LMA, surgical airway) performed in the prehospital setting or within 4 hours of arrival at hospital IV vasopressors administered within 2 hours of arrival at hospital Arrived in the ED with uncontrolled haemorrhage Patient who required EMS initiation of CPR (ie, had a cardiac arrest) during transport, in the ED or within 4 hours of arrival at a hospital
Priority 2	All patients who do not meet the criteria for the other categories are considered priority 2
Priority 3	Discharged from ED with no X-rays or an extremity X-ray that was negative or showed an uncomplicated fracture (ie, a closed extremity fracture without significant displacement or neurovascular compromise); no laboratory testing; received only simple wound repair (single-layer suturing only); and received no medications intravenously (does not include fluids), or inhaled (does not include oxygen) from EMS or in the hospital

These definitions were derived by expert consensus and have been validated in a UK trauma population. Priority 4 (expectant) denotes injuries which are incompatible with life.

CPR, cardiopulmonary resuscitation; EMS, emergency medical services; IV, intravenous; LMA, laryngeal mask airway.

### Input variables selected for modelling

Input variables differ in their complexity and time taken for measurement. Variables that can be readily assessed by first responders in the MI setting were included in the modelling process (summarised in online supplemental table 1). This included all physiological parameters used by existing MI triage tools (first-recorded prehospital HR, RR and systolic BP) with the exception of capillary refill time, which has been found to be a poor reflection of circulatory status and is difficult to measure reliably in challenging settings and in non-white patients.^6 10 19^ In addition to the ability to follow commands (GCS Motor) used by the CareFlight triage tool, all subcomponents of the GCS were included.^6^ However, total GCS score, although known to be an important predictor of outcomes in injured patients, was not included.^7 12^ Total GCS is time-consuming to calculate, with evidence suggesting that scores by paramedics frequently differ from those assigned by emergency physicians; hence, measurement under MI conditions may lack accuracy.^5 19 20^ The ability to breathe spontaneously is an important determinant of outcome and is assessed early within several existing triage tools.^6 10^ TARN does not explicitly record whether patients are spontaneously breathing at the scene of injury, nor does it record the indication for airway interventions.^17^ We assumed that all patients who received an advanced airway intervention at the scene (defined as intubation and ventilation and/or surgical airway and/or the need for airway support) were unable to breathe spontaneously.^10 21^

The presence of injury in anatomical regions including the head, face, chest and limb(s) was included as input variables for modelling using retrospectively calculated Abbreviated Injury Severity (AIS) scores (TARN records AIS based on hospital rather than prehospital data). A binary input (AIS=0, AIS >0) was used rather than a graded assessment of severity. Due to the known difficulties in identifying intra-abdominal injuries based on clinical assessment alone, and the requirement to undertake detailed clinical assessment in order to reliably identify spinal injuries, the presence of spinal and abdominal injuries was not included as input variables.^22 23^ Patient age was dichotomised into age ≥65 years (yes or no), which may be reliably identified by first responders.^12^ Broad injury mechanism (blunt or penetrating) was included.

Input variables described thus far were deemed appropriate for inclusion in both primary and secondary triage tools. Although not conducive to primary triage due to the need for calculation, shock index (HR/systolic BP), which may correlate better with outcome than HR or systolic BP alone, was included in the modelling process as a potential component of a secondary triage tool.^24^

### Model training and internal testing

Four machine learning methods were applied to the model training dataset to distinguish P1 from non-P1 patients. Decision tree (RPART) methodology was included because models can be visualised as bifurcating trees, closely resembling the format of existing primary triage tools. Two other tree-based models with demonstrated value in clinical risk stratification, random forest (RF) and eXtreme Gradient Boosting (XGB), were trained.^25 26^ Further methodological details are presented as online supplemental material. Finally, we included an L1-regularised logistic regression model. We anticipated that non-P1 patients would substantially outnumber P1 patients; hence, we adopted an undersampling strategy to balance the data by leaving out random samples of non-P1 patients.^14^ For each of these models, fivefold cross-validation was applied.^26^

To generate models that were no more complex to apply than existing primary triage tools, modelling included all possible combinations of 3–7 of the available 13 input variables. Model building and selection strategy are summarised in online supplemental figure 2. Models trained using all 13 input variables, although too complex for practical application as triage tools, were also considered as comparators (online supplemental table 2). Additionally, we compared the triage assignments (namely, P1 status) of 10 existing international primary triage tools to the testing dataset (online supplemental table 3).^10^

Previous studies demonstrate that elders (aged 65+ years) are over-represented in the TARN population while constituting 18.3% of the UK population^10^; hence, during testing, we split the TARN testing set by age (ages 16–64 years and 65+ years) to further evaluate model performance.

### Determining feature importance

We assessed the relative importance of individual features (input variables) in model predictions using the TreeSHAP method, a model-agnostic, individualised feature attribution method for explaining predictions.^27^ The resulting Shapley value for a particular feature measures the expected change in model prediction when that feature is present relative to the average model prediction. Additionally, feature importance was estimated by the contribution of each feature to the overall XGB model-predictive performance.^27^

### Selection of models as candidates for primary and secondary triage tools

We sought to identify models that achieved the best possible performance (maximal sensitivity in identifying P1 patients, but also favourable overtriage rate and area under the receiver operating curve (AUC)) across all ages as well as age subgroups, using the minimal number of input variables, to maintain practical applicability. We predetermined that selected models must outperform the best performing existing triage tool, as identified by our study.

In keeping with existing practice, the primary tool candidate was intended to be a paper-based, simple algorithm. The model selected as a secondary tool was adapted into a web-based prototype using the R shiny application.

### External validation of models using the Joint Theatre Trauma Registry database

The UK military’s Joint Theatre Trauma Registry (JTTR) (February 2002–December 2016) was used to externally validate the selected models. JTTR includes consecutive patients who triggered trauma team activation at a deployed medical treatment facility, largely comprising combat casualties during military operations in Iraq and Afghanistan.

Children (<16 years), patients with erroneous data (eg, age over 110 years) and those with injuries recorded as both blunt and penetrating were excluded from the validation (see online supplemental figure 1). As we expected a paucity of prehospital data in this population,^28^ patients’ first recorded hospital physiology was used. Patients with missing data for the input variables were not excluded. Subcomponents of GCS are not routinely recorded within JTTR; these were derived for patients with GCS 15 and unavailable for those with GCS <15. Furthermore, we evaluated candidate models on a subset of JTTR patients with sufficient data to apply the best performing existing tool (subsequently found to be the BCD Triage Sieve), thereby facilitating direct comparison. Triage category definitions were applied as described earlier (table 1): since JTTR does not record the time of interventions, those performed at deployed medical treatment facilities were presumed to have occurred within 4 hours.^28^

### Statistical analyses

Patient characteristics across the model training, internal testing and external validation datasets were compared using the Χ^2^ test (Injury Severity Score (ISS) and age compared using Mann-Whitney U test); p<0.05 was considered statistically significant. Model performance is reported as sensitivity, specificity, undertriage (1-sensitivity) and overtriage (1-positive predictive value). The 95% CIs for the AUC were calculated using deLong’s method (pROC R package, V.1.17.0.1).^29^ The 95% CIs for models’ sensitivity at given specificity points were calculated using 500-stratified bootstrap replicates.^29^

#### Patient and public involvement

Patients or the public were not involved in the design, or conduct, or reporting, or dissemination plans of our research.

## Results

### Training dataset and primary outcome of interest

A total of 200 728 patients were captured by TARN over the 10-year period. After exclusions, the sample consisted of 193 261 patients, of which 21 878 patients (11.3%) fulfilled P1 criteria.

The model training dataset comprised 135 282 patients, with a median age of 64.3 years, in-hospital mortality of 5.7% and predominantly blunt injuries (97%), most commonly low falls (56.3%) (table 2). Patients within the internal test dataset (n=57 979) were older (median age 70.9 years vs 64.3 years, respectively, p<0.001) and more often injured by a low-level fall (62.7% vs 56.3%, p<0.001) compared with patients within the model training dataset.

**Table 2:** Patient and injury characteristics for the model training, testing and external validation cohorts

	Model training dataset (70% TARN: 1 Jan 2008–14 Jul 2016)	Model testing dataset (30% TARN: 15 Jul 2016–31 Dec 2017)	External validation dataset (JTTR: 1 Feb 2002–31 Dec 2016)
Gender			
Male	72 817 (53.8%)	29 532 (50.9%)	5830 (97.9%)
Female	62 465 (46.2%)	28 447 (49.1%)	106 (1.8%)
Missing data	0 (0.0%)	0 (0.0%)	20 (0.3%)
Injury Severity Score			
Median (IQR)	9 (9–16)	9 (9–17)	8 (2–17)
Missing data	0 (0.0%)	0 (0.0%)	13 (0.2%)
Age			
Median (IQR)	64.3 (45.6–82.3)	70.9 (51.6–84.5)	24 (21–28)
16–64 years	69 237 (51.2%)	24 769 (42.7%)	5256 (88.2%)
65+ years	66 045 (48.8%)	33 210 (57.3%)	25 (0.4%)
Missing data	0 (0.0%)	0 (0.0%)	675 (11.3%)
Discharge status			
Alive	127 624 (94.3%)	54 383 (93.8%)	5681 (95.4%)
Dead	7657 (5.7%)	3596 (6.2%)	275 (4.6%)
Missing data	1 (0.0%)	0 (0.0%)	0 (0.0%)
Injury mode			
Blunt	131 208 (97.0%)	56 473 (97.4%)	1092 (18.3%)
Penetrating	4074 (3.0%)	1506 (2.6%)	4864 (81.7%)
Missing data	0 (0.0%)	0 (0.0%)	0 (0.0%)
Injury mechanism			
Fall less than 2 m	76 169 (56.3%)	36 380 (62.7%)	78 (1.3%)
Vehicle incident	30 195 (22.3%)	10 744 (18.5%)	389 (6.5%)
Fall more than 2 m	17 838 (13.2%)	6725 (11.6%)	37 (0.6%)
Blow(s)	4871 (3.6%)	1868 (3.2%)	0 (0.0%)
Stabbing	2871 (2.1%)	1192 (2.1%)	29 (0.5%)
Crush	1065 (0.8%)	268 (0.5%)	76 (1.3%)
Shooting	328 (0.2%)	91 (0.2%)	2316 (38.9%)
Burn	91 (0.07%)	27 (0.05%)	3 (0.1%)
Blast	88 (0.07%)	50 (0.09%)	2926 (49.1%)
Other	1766 (1.3%)	634 (1.1%)	86 (1.4%)
Missing data	0 (0.0%)	0 (0.0%)	16 (0.3%)

JTTR, Joint Theatre Trauma Registry; TARN, Trauma Audit and Research Network.

### Model training and internal testing

In the test set, the BCD Triage Sieve demonstrated the greatest sensitivity at 68% with overtriage at 80.8% (table 3). Existing tools performed less well in the elders’ subgroup compared with younger (16–64 years) adults, with sensitivity 5.8–14.6% lower and overtriage rates 11.5–33.2% higher among elders (online supplemental table 3).

**Table 3:** Performance characteristics of existing triage tools and novel machine learning models among adult patients (16+ years) in the testing (TARN) dataset

	Sensitivity	Specificity	Undertriage	Overtriage	AUC
Existing tools					
BCD Triage Sieve	68.2 (66.9, 69.4)	69.5 (69.1, 69.9)	31.8 (30.6, 33.1)	80.8 (80.2, 81.3)	0.688 (0.682, 0.695)
CareFlight	39.9 (38.6, 41.2)	94.5 (94.3, 94.7)	60.1 (58.8, 61.4)	56.4 (55.0, 57.8)	0.672 (0.666, 0.679)
MPTT-24	48.4 (47.1, 49.7)	66.4 (66.0, 66.8)	51.6 (50.3, 52.9)	86.7 (86.2, 87.2)	0.574 (0.567, 0.581)
MSTART	54.9 (53.6, 56.2)	88.4 (88.1, 88.7)	45.1 (43.8, 46.4)	66.5 (65.5, 67.5)	0.717 (0.710, 0.723)
NARU Triage Sieve	43.0 (41.7, 44.3)	88.3 (88.1, 88.6)	57.0 (55.7, 58.3)	71.8 (70.9, 72.8)	0.657 (0.650, 0.663)
RAMP	37.1 (35.9, 38.4)	94.6 (94.5, 94.8)	62.9 (61.6, 64.1)	57.5 (56.1, 58.9)	0.659 (0.653, 0.665)
Models selected as candidates for novel primary and secondary triage tools					
Primary triage tool candidate (decision tree)	73.0 (71.8, 74.2)	73.9 (73.5, 74.3)	27.0 (25.8, 28.2)	77.0 (76.4, 77.7)	0.782 (0.775, 0.789)
Secondary triage tool candidate (XGB)	77.9 (76.8, 79.0)	73.1 (72.7, 73.5)	22.1 (21.0, 23.2)	76.4 (75.8, 77.0)	0.817 (0.810, 0.824)

Values shown are percentages (except for AUC), accompanied by 95% CIs.

*The best performing model using each method is shown. Both machine learning models and the triage tools were evaluated using the same TARN population (internal testing dataset).

AUC, area under the receiver operating curve; BCD, Battlefield Casualty Drills (UK Military); MPTT-24, Modified Physiological Triage Tool 24 (2017); NARU, National Ambulance Resilience Unit (current UK civilian triage tool); TARN, Trauma Audit and Research Network; XGB, eXtreme Gradient Boosting.

**Figure 1:**
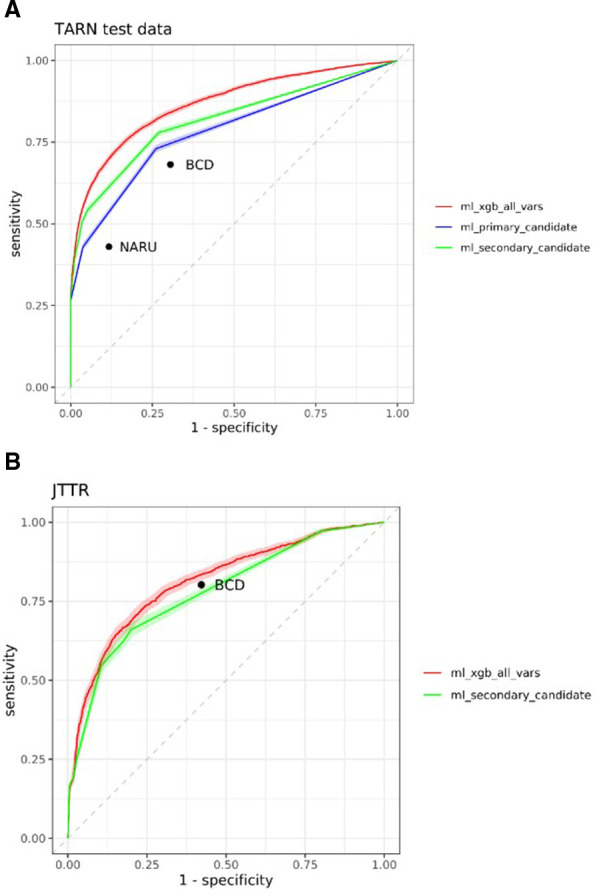
Performance of tool candidate models in the internal and external validation datasets compared with the Battlefield Casualty Drills (BCD) Triage Sieve (best performing existing tool) and the current UK tool, the National Ambulance Resilience Unit (NARU) Triage Sieve. Additionally, the performance of an XGB model using all 13 input variables is shown for comparison (see online supplemental material for more details). JTTR, Joint Theatre Trauma Registry; TARN, Trauma Audit and Research Network; XGB, eXtreme Gradient Boosting.

Four hundred fifty-six models were developed, which, when applied to the internal test dataset, demonstrated greater sensitivity and AUC than all existing tools. Model selection was initially narrowed down to five decision tree models as candidates for primary triage tools and 29 XGB models as candidates for secondary triage tools (see online supplemental figure 2). A comprehensive list, including performance by age subgroups within the internal (TARN) testing and external validation (JTTR) datasets (described later), is detailed in online supplemental table 4A–C. Receiver operating curves demonstrating the performance of the novel primary and secondary tool candidate models when applied to the internal testing dataset are shown in figure 1.

10.1136/emermed-2022-212440.supp2 Supplementary data

### Feature importance

**Figure 2:**
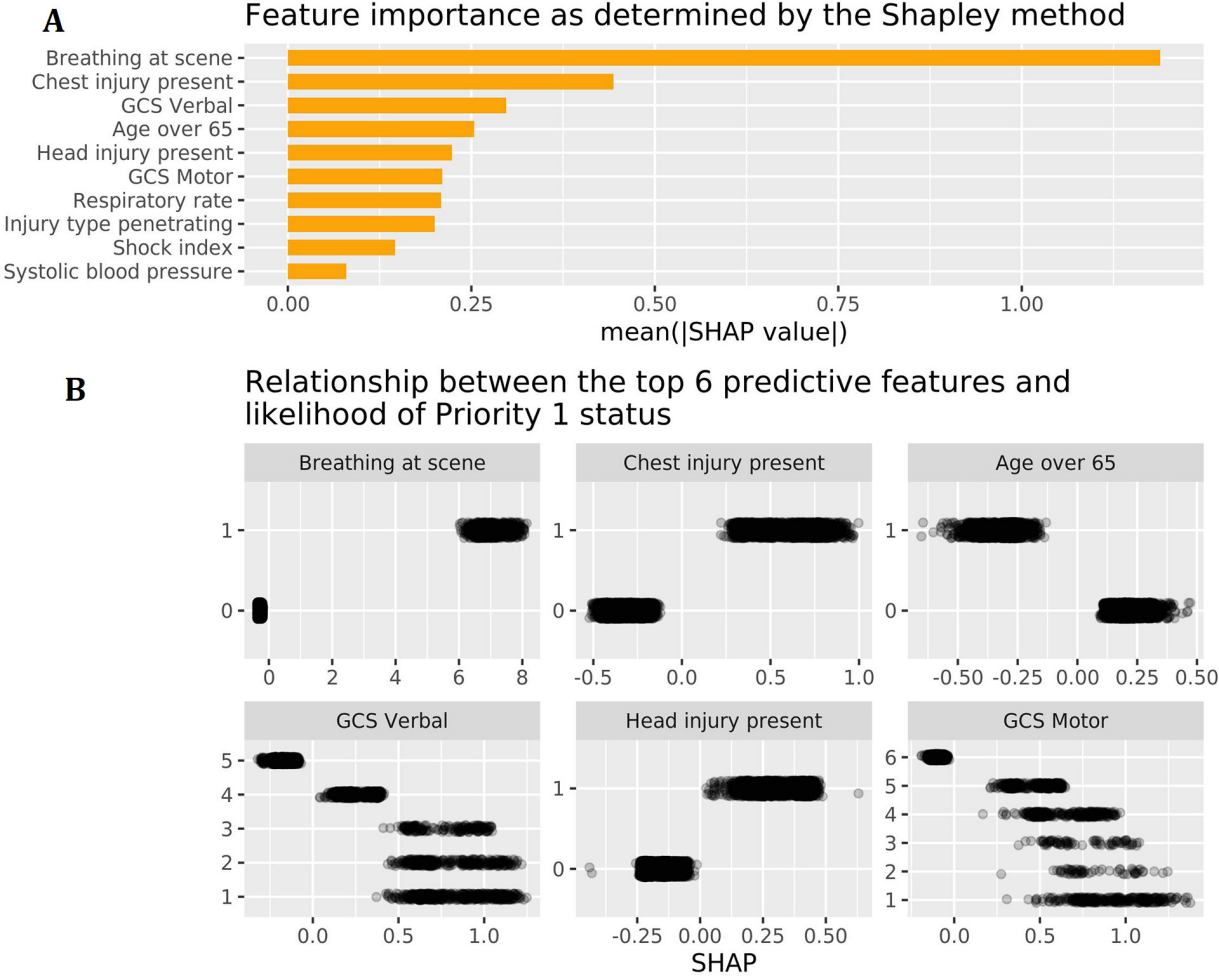
(A) Mean absolute Shapley value for the top 10 predictors. This is followed by the (B) Shapley values for the top six most important features (Shapley values are shown on the x axis, feature values are shown on the y axis). Large, positive Shapley values represent a greater contribution to the likelihood of P1 status. Negative Shapley values represent contributions to non-P1 status. Age over 65 years was found to be negatively predictive of P1 status. GCS Motor, motor subcomponent of the GCS; P1, priority 1.

The top 10 features (figure 2A), and their relative contribution in predicting P1 status (figure 2B) are presented. By far, the most important variable was breathing status (mean Shapley value 1.2), followed by presence of a chest injury and GCS Verbal score. Age >65 years was negatively predictive of P1 status. Any abnormal GCS Verbal or GCS Motor score contributed substantially in predicting P1 status (see figure 2B). The XGB method of determining feature importance yielded similar rankings (online supplemental figure 3).

### Primary and secondary triage tool candidate models

**Figure 3:**
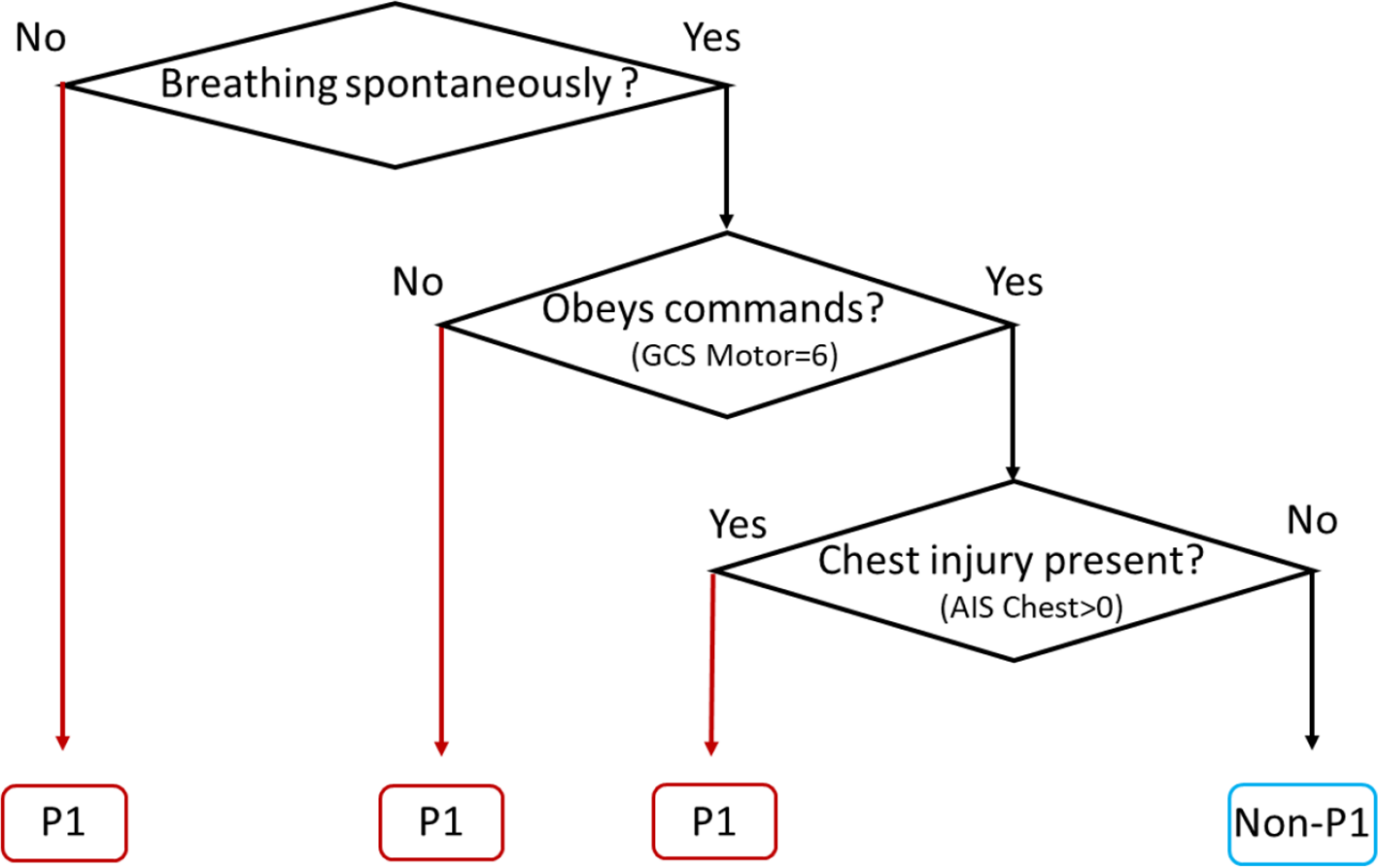
AIS, Abbreviated Injury Severity; P1, priority 1.

The decision tree model selected for clinical adaptation into a primary triage tool (figure 3) used three qualitative binary (yes/no) assessments (breathing status at scene, ability to obey commands, that is, GCS Motor score=6, and presence of a chest injury) to categorise patients as P1 or non-P1. This achieved 73.0% sensitivity, overtriage rate of 77.0% and AUC of 0.782 when applied to the internal testing dataset (see table 3).

**Figure 4:**
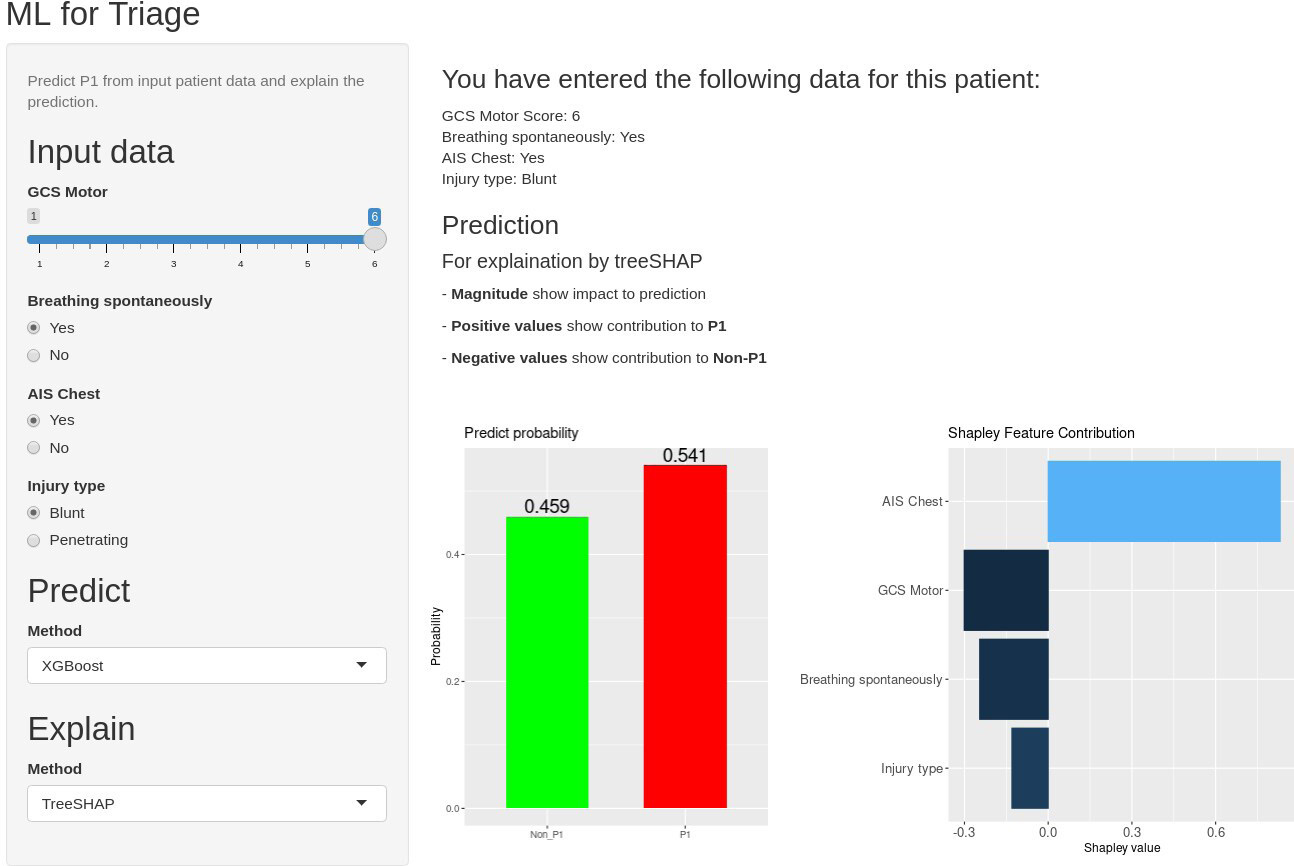
An interactive online application is demonstrated at https://ywxtriageapp.shinyapps.io/mltriage/. AIS, Abbreviated Injury Severity; GCS Motor, motor subcomponent of the GCS; ML, machine learning; P1, priority 1.

The XGB model selected as a secondary triage tool (figure 4) combines four input variables: GCS Motor score, breathing status at scene, presence of chest injury and classification of injury as blunt or penetrating. This model achieved 77.9% sensitivity, overtriage of 76.4% and AUC of 0.817 when applied to the internal testing dataset (figure 1 and table 3). This has been adapted into an online interactive tool (accessible via link: https://ywxtriageapp.shinyapps.io/mltriage/).

### External validation of the secondary triage model (JTTR)

A total of 5956 JTTR patients met inclusion criteria (online supplemental figure 1). Median age was 24 years (IQR 21–28) and most were male (97.9%). Compared with patients in the TARN model training set, JTTR patients had lower mortality (4.6% vs 5.7%, p<0.001) and lower injury severity (median ISS 8 (IQR 2–17) vs median ISS 9 (IQR 9–16), p=0<0.001). A greater proportion of JTTR patients suffered penetrating trauma (81.7% vs 3.0%, p=0<0.001), with high prevalence of blast injury (49.1% vs 0.07%, p=0<0.001) and shooting (38.9% vs 0.2%, p=0<0.001) (see table 2). A total of 2046 (34.3%) JTTR patients had missing GCS Motor scores.

Given the high proportion of JTTR patients missing GCS Motor scores, as well as inability for decision trees to perform predictions when data are missing (unlike XGB and RF), application of the primary tool candidate model to JTTR patients would not reliably measure the model’s external validity. Hence, this was not performed.

Performance of the models shortlisted as candidates for a secondary triage tool for JTTR patients is shown in online supplemental table 4B and model calibration is presented as online supplemental figure 4. The model selected as a secondary tool (XGB model, ID 37) achieved sensitivity of 97.6%, overtriage of 57.5% and AUC of 0.778 (figure 1). Secondary candidate models were evaluated on a subset of JTTR patients containing sufficient data to apply the BCD Triage Sieve (n=5455), thereby facilitating direct comparison (online supplemental table 5): the secondary tool candidate attained comparatively higher sensitivity (97.3% vs 80.2%), but had a higher overtriage rate (58.5% vs 47.4%).

## Discussion

We have developed MI triage tools based on machine learning models that outperform 10 existing international triage tools in predicting the need for time-critical interventions (P1 status) among adults. The best existing primary triage tool, the BCD Triage Sieve, demonstrated sensitivity of 68.2% and overtriage of 80.8% (AUC 0.688), while the selected machine learning primary triage tool achieved a sensitivity of 73% and overtriage of 77% (AUC 0.782). The model selected as a secondary MI triage tool achieved sensitivity of 77.9% and an overtriage rate of 76.4% (AUC 0.817). When externally validated, the secondary tool demonstrated excellent performance with sensitivity of 97.6% and overtriage of 57.5% (AUC 0.778). External validation of the primary tool was precluded by a lack of GCS subcomponent data within the UK combat casualty registry. A novel aspect of this exercise was including anatomical assessment of injuries as part of an MI triage tool and presence of a chest injury was found to be one of the most important variables. Our models serve as evidence-based alternatives to existing tools.

The models proposed are based entirely on qualitative assessments. Eliminating arithmetic calculations (RR and HR) from triage under challenging circumstances has been advocated by expert consensus.^19^ The proposed four-variable secondary tool may also reduce triage time relative to the seven-step NARU and BCD Triage Sieve tools. In addition, decision support using portable device applications has established utility in the MI setting, exemplified by CitizenAID, which enables mutual aid by members of the general public.^30^ Triage using a portable device could help to minimise interuser variability and human error.

Breathing status was the most important predictor of P1 status; this constitutes the opening step in several existing tools.^6^ Our study concurs with the findings of Wallis and Carley, who determined that the GCS Motor component was strongly predictive of P1 status.^31^ The finding that age >65 years is negatively associated with P1 status may be confounded by the predominantly low-risk injury mechanism (low-level falls) in elders in our training dataset: hence, these patients are a poor surrogate for elders injured in an MI. Further work is required to develop effective trauma triage tools for elders, who differ in their physiology, and in whom presence of comorbidities and/or frailty is an important determinant of outcome.^13^ Penetrating mechanism was also an important predictor of P1 status: MIs involving penetrating trauma have historically yielded larger proportions of P1 patients.^5^

A key strength of this study is use of a large sample of injured patients using prospective data collected by trained TARN coordinators.^17^ The primary outcome measure chosen for this study is the only validated outcome measure for MI triage tool performance.^10^ A further strength is that the proposed secondary triage tool has undergone blinded, external validation using the UK military’s JTTR database. This provides estimates of the model’s predictive capability overall, but importantly, also among patients with blast and penetrating mechanisms (under-represented in the TARN dataset) typical of terrorist attacks, the prevalent type of UK MI in recent years.^1^ Selection of an XGB model as a secondary tool, which can make predictions in the context of some missing data, has avoided the possible bias which can result from multiple imputation. Importantly, based on the TARN patients included in our study, both novel tools would generate proportions of P1 casualties that fall within UK national mass casualty planning assumptions.^32^ Notably, no UK or international guidance exists to define acceptable rates of undertriage and overtriage in the major incident setting.

Limitations of this study include use of retrospectively calculated AIS scores (incorporating CT and operative findings) during modelling in place of documented prehospital clinical assessment. While paramedics routinely conduct anatomical assessments during triage in singly injured patients using existing field triage tools and clinical assessment has proven effective in ruling out clinically significant chest injuries, some overtriage can be expected.^12 33^ Clinicians have performed improvised anatomical-based secondary triage following two mass shooting incidents, with a subsequent low rate of undertriage.^5^ Another limitation is the use of singly injured patients within a civilian trauma registry as surrogates for those injured in an MI; outcomes in the MI setting may be worse. Our models focus on predicting P1 status only: however, these patients are at greatest risk of preventable death. In current UK practice, a small proportion of P1 patients may be subsequently assigned P4/expectant status by a senior clinician at scene; this contrasts with practice elsewhere, where triage tools fulfil this role (eg, Australian CareFlight and US START tools).^6 32^ Exclusion of P4 patients (<1% of the sample size) from the modelling process is unlikely to have impacted significantly on study findings. Application of models to the first recorded hospital physiology in JTTR may be biased by prehospital interventions; however, collection of prehospital physiological data during combat is particularly challenging.^28^ The results of external validation in a military trauma population may have limited generalisability to the civilian setting. Further validation of our models in a true MI dataset or a prospective UK civilian database, including blast/penetrating trauma and burns, would provide further assurance of the models’ performance. A further limitation is that we were unable to externally validate our proposed primary tool due to the paucity of prehospital vital signs (GCS) documented in the JTTR dataset.

In conclusion, using machine learning, we developed primary and secondary triage tools which differ from prior tools by incorporating anatomical assessment and have superior sensitivity and more favourable overtriage rates. Although the primary tool requires external validation among patients with injuries similar to those sustained in MI, the proposed secondary triage tool, which was externally validated, may be suitable for use in civilian hospital reception areas and in the military evacuation chain during MIs prior to or in conjunction with senior clinician triage using a portable device.

## Data Availability

Data may be obtained from a third party and are not publicly available. All data relevant to the study are included in the article or uploaded as supplemental information. De-identified patient data used for this study are proprietary to the Trauma Audit and Research Network (TARN), University of Manchester, and may be requested directly from TARN.
