# Supplementary material for: Triage in major incidents: development and external validation of novel machine learning-derived primary and secondary triage tools
Source: Emerg Med J. 2023 Sep 26;41(3):176–83. doi: 10.1136/emermed-2022-212440 (PMC10894820; doi:10.1136/emermed-2022-212440)
Supplement: Supplementary data [file emermed-2022-212440supp001.pdf]

Triage in major incidents: development and external validation of novel machine-learning derived primary and secondary triage tools

Supplementary material

Additional details of machine learning modelling

An overview of study methodology and data processing is presented in Supplementary Figure 1, with a more detailed model development and selection strategy outlined in Supplementary Figure 2.

For the decision tree (also known as Recursive Partitioning And Regression Tree, RPART) method, a limit of a maximum tree depth of 3 was imposed for ease of interpretation.To guard against overfitting, we chose to tune the cost complexity and the tree depth parameter of the decision tree model. Effectively, the tree depth (distance from the root to a terminal node) represents the number of measurables needed in order to determine a triage category. However, we note that unlike triage tools conceived by human experts, it is possible to have the same variable used more than once to split the nodes, if the reuse of variables reduces classification error. A deep tree with many splits tends to overfit the data, and makes it difficult to adapt the model to a tool that can be implemented in practice.

Both random forest (RF) and gradient boosted tree (XGB) are popular machine learning algorithms with strong predictive power. RF is based on averaging an ensemble of trees and the idea of bagging, which lowers the prediction variance. Furthermore, instead of growing each tree using all variables, it randomly chooses a subset of variables at each split of the node in the tree, thereby forcing it to learn through all subsets of available variables. For XGB, the prediction target is estimated by sum-of-trees, and the model is built by successively fitting each tree to the residue of previously fitted trees with no single tree dominating the prediction, while regularizing the fit through multiplication by a scaling factor known as learning rate. In short, XGB estimates the target function by a sum of trees each of which explains a small and different portion of the target and no single tree dominates the prediction.

For the L1-regularized logistic regression model, the penalty parameter, specifying the amount of regularization, was tuned. We add a regularization term in logistic regression so that the solution is well-defined even if the data are perfectly linearly separable.

Initially, models were trained using all 13 input variables (summarised in Supplementary Table 1): the resulting models would be too complex for practical application as tools, but nonetheless act as a useful comparator for model performance (see Supplementary Figure 2 detailing the model building and selection strategy). The optimal hyperparameters that yield the best AUC were selected. For decision tree and logistic regression, a grid search was used; whereas for RF and XGB, random sampling of points in the parameter space was used to try to cover the space as uniformly as possible. For each model, having selected the hyperparameters, a final model was trained on the whole training set (70% of TARN data) and then evaluated on the remaining 30% hold-out data. Models developed using all 13 input variables yielded similar AUC values (range 0.862-0.868, see Supplementary Table 2), except for the decision tree model (AUC 0.782), which also exhibited lower specificity and higher over-triage than the other ML models. All models employing 13 variables attained sensitivity above 72%, exceeding that of the BCD Triage Sieve. Performance characteristics of models employing all 13 input variables were further evaluated by age subgroup (16-64 years and 65+ years (Supplementary Table 2). We note that for ML models evaluated on the 65+ group, while there is slight decrease in AUC compared to the 16-64 group, sensitivity is much worse, except for the decision tree model which has the best sensitivity (66.3%) among all models and triage tools. However, the price of this relatively high sensitivity of decision tree is a high over-triage rate (87.2%).

Existing triage tools were applied to the internal validation dataset to act as comparators to the models proposed as novel triage tools. To overcome the over-representation of elders (65+ years) within the TARN database (see Manuscript, Table 1), who also differ in their physiology to younger adults, tool performance was additionally tested in subgroups by age (16-64 years and 65+ years), as shown in Supplementary Table 3. Existing tools demonstrated lower sensitivity and higher over-triage rates amongst elders compared to younger adults (16-64 years).

We sought to combine the individual models in a weighted fashion by training a super model [1], in which weights are assigned to models based on their predictive power and the final predictions are driven by models with high weights. For the super model, a binomial likelihood maximization using the BFGS quasi-Newton optimization method was used, the model was fitted using the “SuperLearner” R package [2]. The weights are

normalized and sum to one. The super model assigned coefficients (weights) to each individual model, along with the minimized risks. We note that the decision tree model was in fact excluded from the super model, since it has a weight of zero (risk 0.525). The XGB model has highest weight of 0.717 (risk 0.442). Random forest had the second highest weight (0.241, risk 0.454) whilst logistic regression had a low contribution to the overall super model (coefficient 0.041, risk 0.451). The AUC for the super model is 0.868.

The importance of individual features (input parameters) was also estimated using the XGB method (see Supplementary Figure 3). This method yielded similar rankings to those generated by the TREEShap method: breathing status contributed 36% of the total gain, followed by presence of chest injury (13%) and GCS verbal score (11%).

Secondary candidate models were subsequently evaluated on a smaller subset of JTTR patients (n=5455) for which there is complete data available to test the performance of the BCD Triage Sieve, thereby facilitating direct comparison (Supplementary Table 6). The secondary tool candidate (XGB 37) attained comparatively high sensitivity (97.3% vs 80.2%), although this was associated with an 11.1% increase in over-triage (58.5% vs 47.4%).

Supplementary Figure 1: Overview of study methodology and data processing

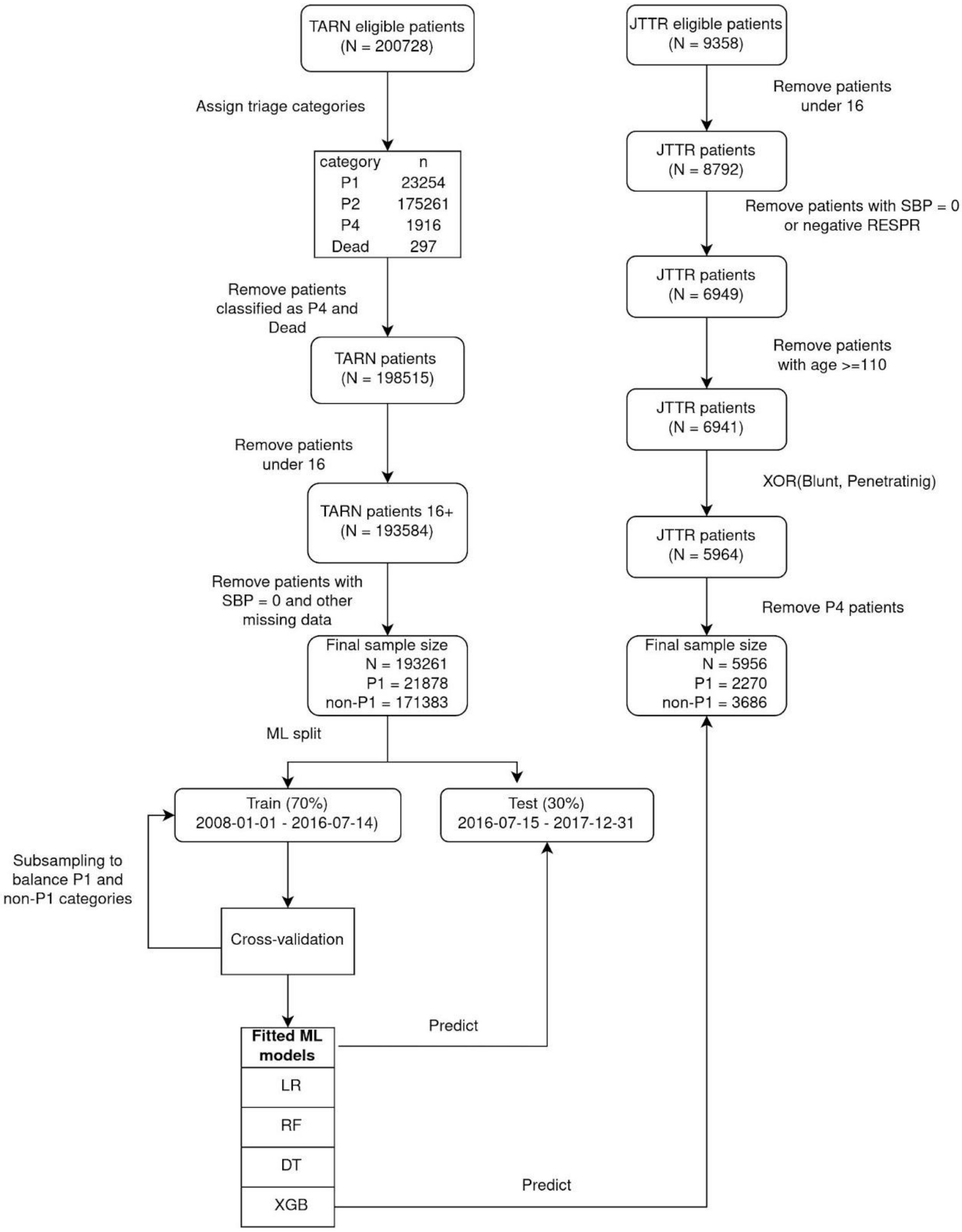

Ledger: Abbreviations: TARN= Trauma Audit and Research Network; JTTR= Joint Theatre Trauma Registry; SBP=Systolic Blood Pressure; XOR=Exclusive/or; LR=Logistic Regression; RF=Random Forest; DT=Decision Tree; XGB= Extreme Gradient Boost; RESPR=Respiratory rate

Supplementary Figure 2: Model building and selection strategy

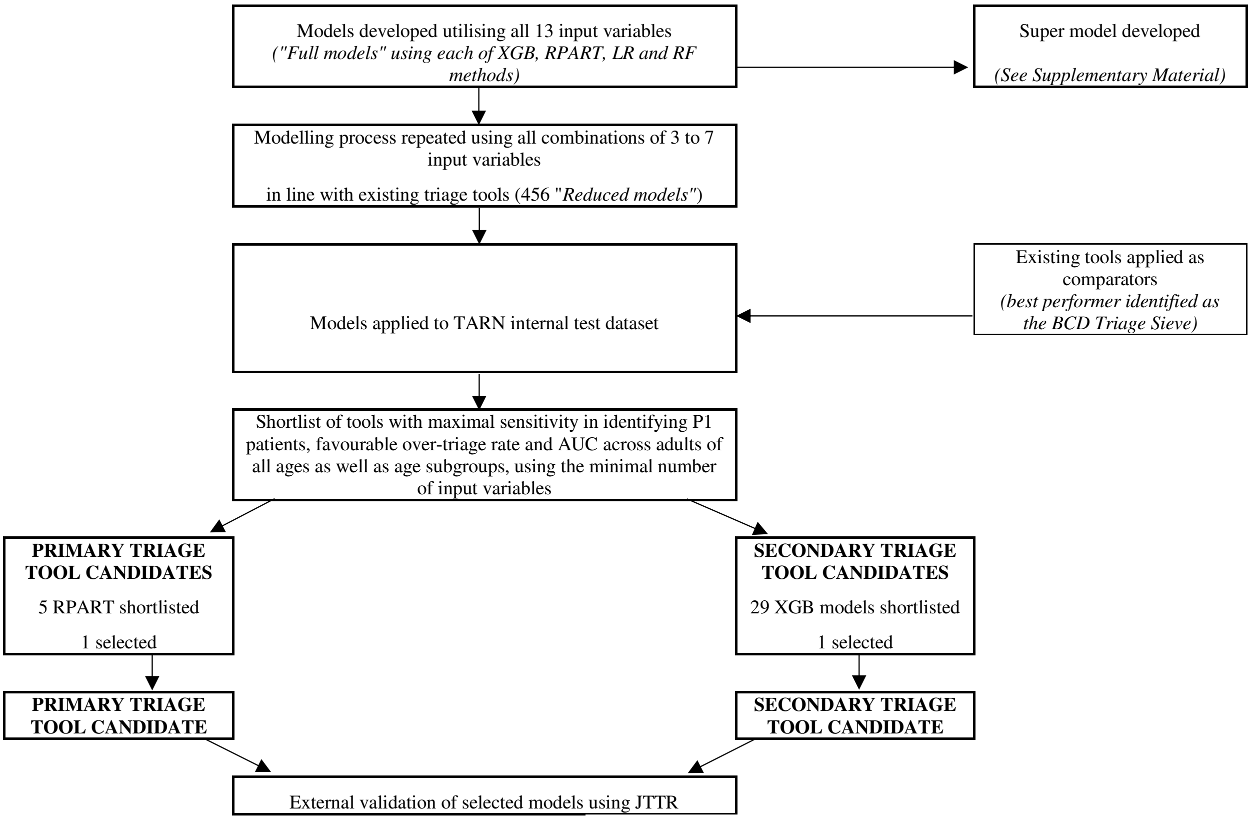

Ledger: XGB=eXtreme Gradient Boosting, RPART=Recursive Partitioning And Regression Trees (i.e. Decision Tree), LR=Logistic regression and RF=random forest, TARN=Trauma Audit and Research Network Registry, JTTR=Joint Theatre Trauma Registry, BCD Triage Sieve=Battlefield Casualty Drills Triage Sieve.

Supplementary Figure 3: Feature importance plot for the XGB model

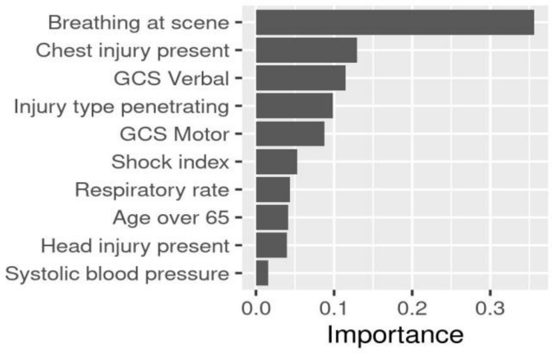

Ledger: Importance of top 10 predictors for the XGB model as measured by the fractional contribution of each feature to the model based on the total gain of each feature's splits. High values represent more predictive features. Respiratory rate is measured in breaths per minute, Systolic blood pressure is measured in mmHg. Presence of chest and head injuries are denoted by a positive Abbreviated Injury Severity score.

Supplementary Figure 4: Calibration plot for models selected as candidate primary and secondary triage tools

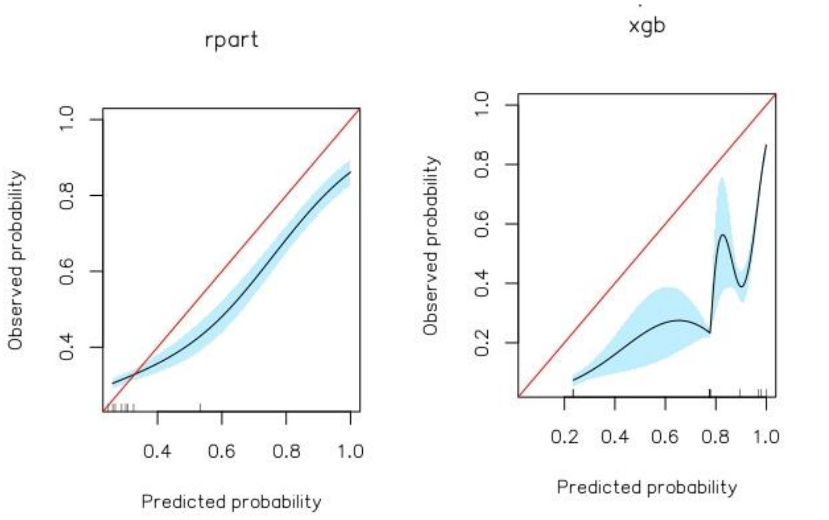

Ledger: calibration plot for the candidate primary (left) and secondary (right) ML models, evaluated using JTTR data.

The calibration curve was estimated by natural splines using the R package gbm [3]. 95% confidence intervals covering 2 standard errors are demonstrated (blue).

For perfect calibration, the calibration curve would align with the 45-degree line (red). It can be seen that the secondary tool (XGB model) over-predicted risk, since the predicted P1 probabilities were greater than the observed probabilities across all patients. This is expected as the secondary tool candidate (XGB model) had high sensitivity but low specificity. In contrast, the calibration curve of the primary tool candidate (decision tree or rpart model) was smoother and the over-prediction was less extreme than XGB, reflecting the fact that the decision tree model had lower sensitivity and higher specificity than XGB.

Supplementary Table 1: Clinical parameters included as input variables for modelling

	Input variables
Physiological parameters*	Heart rate (beats per minute),<br>Respiratory rate (breaths per minute)<br>Systolic blood pressure (mmHg)<br>Ability to breathe spontaneously**<br>GCS Verbal component<br>GCS Motor component<br>GCS Eyes component<br>Shock index***
Anatomical parameters	Presence (AIS>0) or absence (AIS=0) of injury in the following anatomical regions:<br>Head<br>Face<br>Thorax<br>Limb
Age	Age 65 and over (Binary – Yes or No)
Injury Mechanism*	Blunt or penetrating injury

Ledger: GCS=Glasgow Coma Score, AIS=Abbreviated Injury Score. *First recorded pre-hospital physiological parameters and injury mechanism were utilised. **All patients who underwent an advanced airway intervention in the pre-hospital environment were assumed to be unable to breathe. ***Shock index=heart rate/systolic blood pressure.

Supplementary Table 2: Performance of machine learning models utilising all 13 input variables in predicting P1 status amongst patients in the internal (TARN) testing dataset

	Method	Sensitivity	Specificity	Under-triage	Over-triage	AUC
**All adults (16+ years)**	ml_rpart	73.0 [71.8, 74.2]	73.9 [73.5, 74.3]	27.0 [25.8, 28.2]	77.0 [76.4, 77.7]	0.782 [0.775, 0.789]
	ml_rf	72.6 [71.4, 73.8]	86.0 [85.7, 86.3]	27.4 [26.2, 28.6]	64.5 [63.6, 65.4]	0.867 [0.861, 0.873]
	ml_xgb	72.7 [71.5, 73.9]	85.9 [85.6, 86.2]	27.3 [26.1, 28.5]	64.6 [63.7, 65.5]	0.868 [0.862, 0.874]
	ml_lr	72.2 [71.0, 73.4]	85.2 [84.9, 85.5]	27.8 [26.6, 29.0]	65.8 [64.9, 66.6]	0.862 [0.857, 0.868]
**16-64 years subgroup**	ml_rpart	76.0 [74.6, 77.3]	71.8 [71.2, 72.4]	24.0 [22.7, 25.4]	66.8 [65.8, 67.8]	0.794 [0.786, 0.803]
	ml_rf	81.9 [80.7, 83.1]	76.1 [75.5, 76.7]	18.1 [16.9, 19.3]	61.2 [60.2, 62.3]	0.877 [0.871, 0.884]
	ml_xgb	82.3 [81.0, 83.5]	75.7 [75.2, 76.3]	17.7 [16.5, 19.0]	61.5 [60.4, 62.6]	0.879 [0.872, 0.885]
	ml_lr	82.5 [81.2, 83.7]	74.7 [74.1, 75.3]	17.5 [16.3, 18.8]	62.5 [61.4, 63.5]	0.873 [0.866, 0.879]
**65+ years subgroup**	ml_rpart	66.3 [64.0, 68.6]	75.3 [74.8, 75.8]	33.7 [31.4, 36.0]	87.2 [86.5, 87.9]	0.746 [0.733, 0.759]
	ml_rf	51.7 [49.3, 54.1]	92.5 [92.2, 92.8]	48.3 [45.9, 50.7]	72.7 [71.1, 74.2]	0.806 [0.793, 0.818]
	ml_xgb	51.3 [48.9, 53.6]	92.6 [92.3, 92.9]	48.7 [46.4, 51.1]	72.5 [70.9, 74.0]	0.807 [0.795, 0.820]
	ml_lr	49.2 [46.8, 51.5]	92.2 [91.9, 92.5]	50.8 [48.5, 53.2]	74.3 [72.8, 75.8]	0.800 [0.787, 0.812]

Ledger: Results shown are percentages (except for AUC). The best performing model amongst all adults for each method is shown, including performance by age subgroup. Abbreviations: ml=machine learning, rpart= decision tree, rf=random forest, xgb= extreme gradient boosting, lr=logistic regression.

Supplementary Table 3: Performance characteristics of existing triage tools when applied to the internal validation dataset

Tool	Sensitivity	Specificity	Under-triage	Over-triage	AUC
All adults (16+ years)					
BCD Triage Sieve	68.2 [66.9, 69.4]	69.5 [69.1, 69.9]	31.8 [30.6, 33.1]	80.8 [80.2, 81.3]	0.688 [0.682, 0.695]
CareFlight	39.9 [38.6, 41.2]	94.5 [94.3, 94.7]	60.1 [58.8, 61.4]	56.4 [55.0, 57.8]	0.672 [0.666, 0.679]
JumpSTART	42.5 [41.2, 43.8]	92.1 [91.8, 92.3]	57.5 [56.2, 58.8]	63.7 [62.5, 64.9]	0.673 [0.666, 0.679]
MIMMS Triage Sieve	40.5 [39.2, 41.8]	92.0 [91.8, 92.3]	59.5 [58.2, 60.8]	64.9 [63.7, 66.1]	0.663 [0.656, 0.669]
MPTT	50.5 [49.2, 51.8]	62.4 [62.0, 62.8]	49.5 [48.2, 50.8]	87.5 [87.1, 87.9]	0.565 [0.558, 0.571]
MPTT-24	48.4 [47.1, 49.7]	66.4 [66.0, 66.8]	51.6 [50.3, 52.9]	86.7 [86.2, 87.2]	0.574 [0.567, 0.581]
MSTART	54.9 [53.6, 56.2]	88.4 [88.1, 88.7]	45.1 [43.8, 46.4]	66.5 [65.5, 67.5]	0.717 [0.710, 0.723]
NARU Triage Sieve	43.0 [41.7, 44.3]	88.3 [88.1, 88.6]	57.0 [55.7, 58.3]	71.8 [70.9, 72.8]	0.657 [0.650, 0.663]
RAMP	37.1 [35.9, 38.4]	94.6 [94.5, 94.8]	62.9 [61.6, 64.1]	57.5 [56.1, 58.9]	0.659 [0.653, 0.665]
START	51.8 [50.5, 53.2]	90.0 [89.7, 90.2]	48.2 [46.8, 49.5]	64.5 [63.5, 65.6]	0.709 [0.702, 0.716]
16-64 years subgroup					
BCD Triage Sieve	72.7 [71.2, 74.1]	64.8 [64.2, 65.5]	27.3 [25.9, 28.8]	72.4 [71.5, 73.3]	0.687 [0.680, 0.695]
CareFlight	42.7 [41.1, 44.3]	94.3 [94.0, 94.6]	57.3 [55.7, 58.9]	42.0 [40.2, 43.8]	0.685 [0.677, 0.693]
JumpSTART	45.5 [43.9, 47.0]	91.2 [90.9, 91.6]	54.5 [53.0, 56.1]	51.1 [49.4, 52.7]	0.684 [0.675, 0.692]
MIMMS Triage Sieve	43.0 [41.4, 44.6]	92.6 [92.2, 93.0]	57.0 [55.4, 58.6]	48.2 [46.5, 50.0]	0.678 [0.670, 0.686]
MPTT	52.3 [50.7, 53.9]	57.1 [56.4, 57.8]	47.7 [46.1, 49.3]	81.6 [80.9, 82.4]	0.547 [0.538, 0.555]
MPTT-24	50.5 [48.9, 52.1]	61.6 [60.9, 62.2]	49.5 [47.9, 51.1]	80.5 [79.7, 81.3]	0.560 [0.552, 0.569]
MSTART	57.6 [56.1, 59.2]	88.9 [88.5, 89.3]	42.4 [40.8, 43.9]	51.0 [49.6, 52.5]	0.733 [0.725, 0.741]
NARU Triage Sieve	47.1 [45.5, 48.7]	87.5 [87.0, 87.9]	52.9 [51.3, 54.5]	59.0 [57.6, 60.5]	0.673 [0.665, 0.681]
RAMP	39.6 [38.1, 41.2]	94.4 [94.1, 94.7]	60.4 [58.8, 61.9]	43.4 [41.5, 45.3]	0.670 [0.662, 0.678]
START	54.4 [52.8, 55.9]	90.7 [90.3, 91.1]	45.6 [44.1, 47.2]	48.1 [46.5, 49.6]	0.725 [0.717, 0.733]
65+ years subgroup					
BCD Triage Sieve	58.1 [55.7, 60.4]	72.6 [72.1, 73.1]	41.9 [39.6, 44.3]	89.6 [89.0, 90.2]	0.653 [0.642, 0.665]
CareFlight	33.7 [31.4, 36.0]	94.6 [94.4, 94.9]	66.3 [64.0, 68.6]	74.5 [72.6, 76.3]	0.642 [0.630, 0.653]
JumpSTART	35.8 [33.5, 38.1]	92.6 [92.3, 92.9]	64.2 [61.9, 66.5]	79.1 [77.6, 80.6]	0.642 [0.630, 0.653]
MIMMS Triage Sieve	34.9 [32.6, 37.2]	91.7 [91.3, 92.0]	65.1 [62.8, 67.4]	81.4 [80.0, 82.8]	0.633 [0.621, 0.644]
MPTT	46.5 [44.1, 48.9]	65.9 [65.4, 66.5]	53.5 [51.1, 55.9]	93.1 [92.6, 93.5]	0.562 [0.550, 0.574]
MPTT-24	43.7 [41.3, 46.1]	69.6 [69.1, 70.1]	56.3 [53.9, 58.7]	92.7 [92.2, 93.2]	0.567 [0.555, 0.579]
MSTART	48.9 [46.5, 51.3]	88.0 [87.7, 88.4]	51.1 [48.7, 53.5]	81.8 [80.6, 82.9]	0.685 [0.673, 0.697]
NARU Triage Sieve	33.7 [31.5, 36.0]	88.9 [88.6, 89.3]	66.3 [64.0, 68.5]	85.8 [84.7, 86.8]	0.613 [0.602, 0.625]
RAMP	31.6 [29.4, 33.8]	94.8 [94.6, 95.1]	68.4 [66.2, 70.6]	75.1 [73.2, 76.9]	0.632 [0.621, 0.643]
START	46.2 [43.8, 48.6]	89.5 [89.1, 89.8]	53.8 [51.4, 56.2]	80.7 [79.5, 81.9]	0.678 [0.666, 0.690]

Ledger: BCD Triage Sieve=Battlefield Casualty Drills Triage Sieve (UK Military), CareFlight (Australia), JumpSTART (US paediatric triage tool), MIMMS Triage Sieve=Major Incident Medical Management and Support Triage Sieve, MPTT=Modified Physiological Triage Tool (tool modelled in UK military casualties), MPTT-24 (modification of MPTT, 2017), START=Simple Triage and Rapid Treatment (US adult tool), MSTART=modified START, NARU Triage Sieve=National Ambulance Resilience Unit Triage Sieve (Current UK civilian tool), RAMP=Rapid Assessment of Mentation and Pulse (New York Fire Department).

Supplementary Tables 4A-C: See landscape format document

Supplementary Table 5: External validation of shortlisted models and the Battlefield Casualty Drills Triage Sieve (comparator) using the Joint Theatre Trauma Registry (n=5455)

Method	Sensitivity	Specificity	Under-triage	Over-triage	AUC
**Comparator (best existing tool):**					
BCD Triage Sieve	0.802 [0.784, 0.819]	0.578 [0.561, 0.595]	0.198 [0.181, 0.216]	0.474 [0.456, 0.492]	0.690 [0.678, 0.702]
**Primary tool candidate models**					
rpart_1	0.330 [0.310, 0.351]	0.892 [0.881, 0.902]	0.670 [0.649, 0.690]	0.360 [0.331, 0.390]	0.618 [0.606, 0.629]
rpart_3	0.330 [0.310, 0.351]	0.892 [0.881, 0.902]	0.670 [0.649, 0.690]	0.360 [0.331, 0.390]	0.618 [0.607, 0.630]
rpart_37	0.330 [0.310, 0.351]	0.892 [0.881, 0.902]	0.670 [0.649, 0.690]	0.360 [0.331, 0.390]	0.618 [0.607, 0.630]
rpart_52	0.479 [0.457, 0.501]	0.752 [0.737, 0.766]	0.521 [0.499, 0.543]	0.470 [0.447, 0.494]	0.611 [0.598, 0.624]
rpart_124	0.437 [0.415, 0.459]	0.879 [0.868, 0.890]	0.563 [0.541, 0.585]	0.322 [0.297, 0.348]	0.668 [0.656, 0.680]
**Secondary tool candidate models**					
xgb_1	0.667 [0.646, 0.688]	0.788 [0.774, 0.802]	0.333 [0.312, 0.354]	0.353 [0.332, 0.374]	0.755 [0.743, 0.768]
xgb_3	0.667 [0.646, 0.688]	0.788 [0.774, 0.802]	0.333 [0.312, 0.354]	0.353 [0.332, 0.374]	0.755 [0.743, 0.768]
xgb_37	0.973 [0.964, 0.979]	0.199 [0.186, 0.213]	0.027 [0.021, 0.036]	0.585 [0.571, 0.599]	0.780 [0.768, 0.792]
xgb_38	0.667 [0.646, 0.688]	0.788 [0.774, 0.802]	0.333 [0.312, 0.354]	0.353 [0.332, 0.374]	0.755 [0.743, 0.768]
xgb_41	0.667 [0.646, 0.688]	0.788 [0.774, 0.802]	0.333 [0.312, 0.354]	0.353 [0.332, 0.374]	0.782 [0.769, 0.795]
xgb_42	0.667 [0.646, 0.688]	0.788 [0.774, 0.802]	0.333 [0.312, 0.354]	0.353 [0.332, 0.374]	0.748 [0.734, 0.761]
xgb_43	0.675 [0.654, 0.695]	0.791 [0.777, 0.805]	0.325 [0.305, 0.346]	0.347 [0.326, 0.367]	0.776 [0.762, 0.790]
xgb_44	0.973 [0.964, 0.979]	0.199 [0.186, 0.213]	0.027 [0.021, 0.036]	0.585 [0.571, 0.599]	0.780 [0.768, 0.792]
xgb_53	0.667 [0.646, 0.688]	0.788 [0.774, 0.802]	0.333 [0.312, 0.354]	0.353 [0.332, 0.374]	0.748 [0.734, 0.761]
xgb_54	0.675 [0.654, 0.695]	0.791 [0.777, 0.805]	0.325 [0.305, 0.346]	0.347 [0.326, 0.367]	0.777 [0.763, 0.790]
xgb_121	0.973 [0.964, 0.979]	0.199 [0.186, 0.213]	0.027 [0.021, 0.036]	0.585 [0.571, 0.599]	0.780 [0.768, 0.792]
xgb_124	0.975 [0.966, 0.981]	0.174 [0.162, 0.187]	0.025 [0.019, 0.034]	0.592 [0.578, 0.606]	0.777 [0.764, 0.790]
xgb_125	0.973 [0.964, 0.979]	0.199 [0.186, 0.213]	0.027 [0.021, 0.036]	0.585 [0.571, 0.599]	0.778 [0.766, 0.790]
xgb_130	0.667 [0.646, 0.688]	0.788 [0.774, 0.802]	0.333 [0.312, 0.354]	0.353 [0.332, 0.374]	0.748 [0.734, 0.761]
xgb_131	0.674 [0.653, 0.695]	0.793 [0.779, 0.807]	0.326 [0.305, 0.347]	0.344 [0.324, 0.365]	0.774 [0.760, 0.788]
xgb_141	0.676 [0.655, 0.696]	0.792 [0.778, 0.805]	0.324 [0.304, 0.345]	0.346 [0.325, 0.367]	0.768 [0.754, 0.782]
xgb_154	0.965 [0.955, 0.972]	0.171 [0.159, 0.184]	0.035 [0.028, 0.045]	0.596 [0.582, 0.610]	0.695 [0.680, 0.709]
xgb_165	0.684 [0.663, 0.704]	0.792 [0.778, 0.805]	0.316 [0.296, 0.337]	0.343 [0.323, 0.364]	0.788 [0.774, 0.801]
xgb_166	0.676 [0.655, 0.696]	0.791 [0.777, 0.805]	0.324 [0.304, 0.345]	0.346 [0.326, 0.367]	0.770 [0.757, 0.784]
xgb_250	0.973 [0.964, 0.979]	0.199 [0.186, 0.213]	0.027 [0.021, 0.036]	0.585 [0.571, 0.599]	0.781 [0.769, 0.794]
xgb_251	0.972 [0.964, 0.979]	0.203 [0.190, 0.217]	0.028 [0.021, 0.036]	0.584 [0.570, 0.599]	0.792 [0.779, 0.804]
xgb_270	0.722 [0.702, 0.742]	0.701 [0.685, 0.716]	0.278 [0.258, 0.298]	0.416 [0.396, 0.435]	0.785 [0.772, 0.798]
xgb_271	0.670 [0.649, 0.691]	0.800 [0.786, 0.813]	0.330 [0.309, 0.351]	0.338 [0.318, 0.359]	0.770 [0.756, 0.784]
xgb_289	0.971 [0.962, 0.977]	0.195 [0.182, 0.209]	0.029 [0.023, 0.038]	0.587 [0.573, 0.601]	0.778 [0.765, 0.791]
xgb_311	0.721 [0.701, 0.741]	0.704 [0.689, 0.719]	0.279 [0.259, 0.299]	0.413 [0.393, 0.433]	0.781 [0.768, 0.795]
xgb_380	0.971 [0.962, 0.977]	0.196 [0.183, 0.210]	0.029 [0.023, 0.038]	0.587 [0.573, 0.601]	0.795 [0.782, 0.807]
xgb_392	0.976 [0.968, 0.982]	0.174 [0.162, 0.187]	0.024 [0.018, 0.032]	0.592 [0.578, 0.606]	0.800 [0.788, 0.812]
xgb_402	0.709 [0.688, 0.729]	0.732 [0.717, 0.747]	0.291 [0.271, 0.312]	0.394 [0.374, 0.414]	0.783 [0.769, 0.796]
xgb_417	0.976 [0.968, 0.982]	0.181 [0.168, 0.194]	0.024 [0.018, 0.032]	0.590 [0.576, 0.604]	0.799 [0.787, 0.812]

Ledger: *this is a reduced JTTR dataset for which a complete set of physiological data exists for application of the BCD Triage Sieve (the best performing existing tool, selected as a comparator) can be applied. To allow direct comparison, the models shortlisted as candidates for triage tools are applied to the reduced dataset. rpart=decision tree (Recursive Partitioning And Regression Trees), xgb= extreme gradient boosting.

References:

[1] Van der Laan, Mark J., Eric C. Polley, and Alan E. Hubbard. "Super learner." *Statistical applications in genetics and molecular biology* 6.1 (2007).

[2] Eric Polley, Erin LeDell, Chris Kennedy and Mark van der Laan (2019). SuperLearner: Super Learner Prediction. R package version 2.0-26. https://CRAN.R-project.org/package=SuperLearner

[3] Brandon Greenwell, Bradley Boehmke, Jay Cunningham and GBM Developers (2022). gbm: Generalized Boosted Regression Models. R package version 2.1.8.1. https://CRAN.R-project.org/package=gbm].
