# Supplementary material for: Triage in major incidents: development and external validation of novel machine learning-derived primary and secondary triage tools
Source: Emerg Med J. 2023 Sep 26;41(3):176–83. doi: 10.1136/emermed-2022-212440 (PMC10894820; doi:10.1136/emermed-2022-212440)
Supplement: Supplementary data [file emermed-2022-212440supp002.pdf]

Supplementary Table 4A: Models shortlisted as candidates for primary and secondary tools: Description of model variables

Task ID	Model variables	Variables
	**Candidate primary tools (Decision Tree (RPART) models)**	
1	Breathing status at scene|Chest injury present|GCS Motor Score	3
3	Breathing status at scene|Chest injury present|GCS Verbal Score	3
37	Breathing status at scene|Chest injury present|GCS Motor Score|Injury type	4
52	Breathing status at scene|Chest injury present|GCS Verbal Score|Respiratory rate	4
124	Breathing status at scene|Chest injury present|GCS Motor Score|Injury type|Respiratory rate	5
	**Candidate secondary tools (Extreme Gradient Boosting (XGB) models)**	
1	Breathing status at scene|Chest injury present|GCS Motor Score	3
3	Breathing status at scene|Chest injury present|GCS Verbal Score	3
37	Breathing status at scene|Chest injury present|GCS Motor Score|Injury type	4
38	Breathing status at scene|Chest injury present|GCS Motor Score|GCS Verbal Score	4
41	Breathing status at scene|Chest injury present|GCS Motor Score|Respiratory rate	4
42	Breathing status at scene|Chest injury present|GCS Motor Score|Head injury present	4
43	Breathing status at scene|Chest injury present|GCS Motor Score|Systolic blood pressure	4
44	Breathing status at scene|Chest injury present|Injury type|GCS Verbal Score	4
53	Breathing status at scene|Chest injury present|GCS Verbal Score|Head injury present	4
54	Breathing status at scene|Chest injury present|GCS Verbal Score|Systolic blood pressure	4
121	Breathing status at scene|Chest injury present|GCS Motor Score|Injury type|GCS Verbal Score	5
124	Breathing status at scene|Chest injury present|GCS Motor Score|Injury type|Respiratory rate	5
125	Breathing status at scene|Chest injury present|GCS Motor Score|Injury type|Head injury present	5
130	Breathing status at scene|Chest injury present|GCS Motor Score|GCS Verbal Score|Head injury present	5
131	Breathing status at scene|Chest injury present|GCS Motor Score|GCS Verbal Score|Systolic blood pressure	5
141	Breathing status at scene|Chest injury present|GCS Motor Score|Head injury present|Systolic blood pressure	5
154	Breathing status at scene|Chest injury present|Injury type|Respiratory rate|Head injury present	5
165	Breathing status at scene|Chest injury present|GCS Verbal Score|Respiratory rate|Systolic blood pressure	5
166	Breathing status at scene|Chest injury present|GCS Verbal Score|Head injury present|Systolic blood pressure	5
250	Breathing status at scene|Chest injury present|GCS Motor Score|Injury type|GCS Verbal Score|Head injury present	6
251	Breathing status at scene|Chest injury present|GCS Motor Score|Injury type|GCS Verbal Score|Systolic blood pressure	6
270	Breathing status at scene|Chest injury present|GCS Motor Score|GCS Verbal Score|Respiratory rate|Systolic blood pressure	6
271	Breathing status at scene|Chest injury present|GCS Motor Score|GCS Verbal Score|Head injury present|Systolic blood pressure	6
289	Breathing status at scene|Chest injury present|Injury type|GCS Verbal Score|Respiratory rate|Head injury present	6
311	Breathing status at scene|Chest injury present|GCS Verbal Score|Respiratory rate|Head injury present|Systolic blood pressure	6
380	Breathing status at scene|Chest injury present|GCS Motor Score|Injury type|GCS Verbal Score|Respiratory rate|Head injury present	7
392	Breathing status at scene|Chest injury present|GCS Motor Score|Injury type|Respiratory rate|Head injury present|Systolic blood pressure	7
402	Breathing status at scene|Chest injury present|GCS Motor Score|GCS Verbal Score|Respiratory rate|Head injury present|Systolic blood pressure	7
417	Breathing status at scene|Chest injury present|Injury type|GCS Verbal Score|Respiratory rate|Head injury present|Systolic blood pressure	7

Supplementary Table 4B: Performance characteristics of models shortlisted as primary and secondary tool candidates

	**Internal validation using TARN testing dataset (all adult patients)**					**External validation in JTTR (n=5956)**				
**Task ID**	**Sensitivity**	**Specificity**	**Under-triage**	**Over-triage**	**AUC**	**Sensitivity**	**Specificity**	**Under-triage**	**Over-triage**	**AUC**
RPART:										
1	73·0 [71·8, 74·2]	73·9 [73·5, 74·3]	27·0 [25·8, 28·2]	77·0 [76·4, 77·7]	0·772 [0·765, 0·779]	34·0 [32·1, 36·0]	89·0 [87·9, 90·0]	66·0 [64·0, 67·9]	34·4 [31·7, 37·2]	0·622 [0·611, 0·633]
3	72·3 [71·1, 73·5]	74·5 [74·1, 74·8]	27·7 [26·5, 28·9]	76·9 [76·2, 77·5]	0·775 [0·768, 0·782]	34·0 [32·1, 36·0]	89·0 [87·9, 90·0]	66·0 [64·0, 67·9]	34·4 [31·7, 37·2]	0·623 [0·612, 0·634]
37	73·0 [71·8, 74·2]	73·9 [73·5, 74·3]	27·0 [25·8, 28·2]	77·0 [76·4, 77·7]	0·782 [0·775, 0·789]	49·8 [47·7, 51·9]	74·5 [73·0, 75·9]	50·2 [48·1, 52·3]	45·4 [43·3, 47·6]	0·616 [0·604, 0·629]
52	72·3 [71·1, 73·5]	74·5 [74·1, 74·8]	27·7 [26·5, 28·9]	76·9 [76·2, 77·5]	0·780 [0·773, 0·787]	34·0 [32·1, 36·0]	89·0 [87·9, 90·0]	66·0 [64·0, 67·9]	34·4 [31·7, 37·2]	0·623 [0·612, 0·634]
124	73·0 [71·8, 74·2]	73·9 [73·5, 74·3]	27·0 [25·8, 28·2]	77·0 [76·4, 77·7]	0·777 [0·770, 0·783]	46·1 [44·0, 48·2]	86·3 [85·2, 87·4]	53·9 [51·8, 56·0]	32·5 [30·2, 34·9]	0·671 [0·659, 0·683]
XGB:										
1	73·0 [71·8, 74·2]	73·9 [73·5, 74·3]	27·0 [25·8, 28·2]	77·0 [76·4, 77·7]	0·783 [0·776, 0·790]	70·5 [68·6, 72·4]	73·7 [72·2, 75·1]	29·5 [27·6, 31·4]	37·7 [35·9, 39·6]	0·754 [0·742, 0·766]
3	72·3 [71·1, 73·5]	74·5 [74·1, 74·8]	27·7 [26·5, 28·9]	76·9 [76·2, 77·5]	0·796 [0·789, 0·803]	70·5 [68·6, 72·4]	73·7 [72·2, 75·1]	29·5 [27·6, 31·4]	37·7 [35·9, 39·6]	0·754 [0·742, 0·766]
37	77·9 [76·8, 79·0]	73·1 [72·7, 73·5]	22·1 [21·0, 23·2]	76·4 [75·8, 77·0]	0·817 [0·810, 0·824]	97·6 [96·8, 98·2]	18·6 [17·4, 19·9]	2·4 [1·8, 3·2]	57·5 [56·2, 58·9]	0·778 [0·766, 0·790]
38	73·8 [72·6, 75·0]	73·6 [73·2, 73·9]	26·2 [25·0, 27·4]	77·1 [76·5, 77·7]	0·798 [0·792, 0·805]	70·5 [68·6, 72·4]	73·7 [72·2, 75·1]	29·5 [27·6, 31·4]	37·7 [35·9, 39·6]	0·754 [0·742, 0·766]
41	73·0 [71·8, 74·2]	73·9 [73·5, 74·3]	27·0 [25·8, 28·2]	77·0 [76·4, 77·7]	0·808 [0·801, 0·815]	70·5 [68·6, 72·4]	73·7 [72·2, 75·1]	29·5 [27·6, 31·4]	37·7 [35·9, 39·6]	0·779 [0·767, 0·792]
42	73·0 [71·8, 74·2]	73·9 [73·5, 74·3]	27·0 [25·8, 28·2]	77·0 [76·4, 77·7]	0·798 [0·791, 0·805]	70·5 [68·6, 72·4]	73·7 [72·2, 75·1]	29·5 [27·6, 31·4]	37·7 [35·9, 39·6]	0·749 [0·736, 0·762]
43	70·0 [68·7, 71·2]	78·5 [78·2, 78·9]	30·0 [28·8, 31·3]	74·3 [73·6, 75·0]	0·809 [0·802, 0·816]	71·2 [69·3, 73·1]	74·0 [72·5, 75·4]	28·8 [26·9, 30·7]	37·2 [35·4, 39·1]	0·775 [0·762, 0·788]
44	77·3 [76·2, 78·4]	73·6 [73·3, 74·0]	22·7 [21·6, 23·8]	76·2 [75·6, 76·8]	0·830 [0·824, 0·837]	97·6 [96·8, 98·2]	18·6 [17·4, 19·9]	2·4 [1·8, 3·2]	57·5 [56·2, 58·9]	0·778 [0·766, 0·790]
53	72·3 [71·1, 73·5]	74·5 [74·1, 74·8]	27·7 [26·5, 28·9]	76·9 [76·2, 77·5]	0·802 [0·795, 0·809]	70·5 [68·6, 72·4]	73·7 [72·2, 75·1]	29·5 [27·6, 31·4]	37·7 [35·9, 39·6]	0·749 [0·736, 0·762]
54	70·7 [69·5, 71·9]	77·7 [77·4, 78·1]	29·3 [28·1, 30·5]	74·7 [74·0, 75·4]	0·816 [0·810, 0·823]	71·2 [69·3, 73·1]	74·0 [72·5, 75·4]	28·8 [26·9, 30·7]	37·2 [35·4, 39·1]	0·776 [0·763, 0·789]
121	78·7 [77·6, 79·8]	72·7 [72·4, 73·1]	21·3 [20·2, 22·4]	76·5 [75·9, 77·1]	0·833 [0·827, 0·839]	97·6 [96·8, 98·2]	18·6 [17·4, 19·9]	2·4 [1·8, 3·2]	57·5 [56·2, 58·9]	0·778 [0·766, 0·790]
124	71·8 [70·6, 73·0]	81·9 [81·6, 82·2]	28·2 [27·0, 29·4]	70·3 [69·5, 71·1]	0·834 [0·827, 0·841]	97·8 [97·0, 98·3]	16·3 [15·1, 17·5]	2·2 [1·7, 3·0]	58·2 [56·8, 59·5]	0·775 [0·763, 0·787]
125	77·6 [76·5, 78·7]	73·6 [73·2, 74·0]	22·4 [21·3, 23·5]	76·2 [75·5, 76·8]	0·836 [0·829, 0·842]	97·6 [96·8, 98·2]	18·6 [17·4, 19·9]	2·4 [1·8, 3·2]	57·5 [56·2, 58·9]	0·778 [0·766, 0·790]
130	73·8 [72·6, 74·9]	73·6 [73·2, 74·0]	26·2 [25·1, 27·4]	77·1 [76·5, 77·7]	0·804 [0·797, 0·811]	70·5 [68·6, 72·4]	73·7 [72·2, 75·1]	29·5 [27·6, 31·4]	37·7 [35·9, 39·6]	0·749 [0·736, 0·762]
131	71·3 [70·1, 72·5]	77·7 [77·4, 78·1]	28·7 [27·5, 29·9]	74·6 [73·9, 75·3]	0·819 [0·812, 0·826]	71·2 [69·3, 73·0]	74·2 [72·7, 75·6]	28·8 [27·0, 30·7]	37·1 [35·2, 39·0]	0·771 [0·758, 0·783]
141	71·0 [69·8, 72·2]	77·9 [77·5, 78·2]	29·0 [27·8, 30·2]	74·6 [73·9, 75·2]	0·819 [0·813, 0·826]	71·3 [69·4, 73·2]	74·0 [72·6, 75·4]	28·7 [26·8, 30·6]	37·2 [35·3, 39·1]	0·768 [0·755, 0·781]
154	71·3 [70·1, 72·5]	78·0 [77·7, 78·4]	28·7 [27·5, 29·9]	74·4 [73·7, 75·0]	0·829 [0·823, 0·835]	96·7 [95·9, 97·4]	17·1 [15·9, 18·4]	3·3 [2·6, 4·1]	58·2 [56·8, 59·5]	0·702 [0·688, 0·715]
165	68·7 [67·4, 69·9]	81·7 [81·3, 82·0]	31·3 [30·1, 32·6]	71·5 [70·7, 72·3]	0·826 [0·820, 0·833]	72·0 [70·1, 73·9]	74·0 [72·6, 75·4]	28·0 [26·1, 29·9]	36·9 [35·1, 38·8]	0·784 [0·772, 0·797]
166	71·9 [70·7, 73·1]	76·2 [75·9, 76·6]	28·1 [26·9, 29·3]	75·7 [75·0, 76·3]	0·822 [0·815, 0·828]	71·3 [69·4, 73·2]	74·0 [72·5, 75·4]	28·7 [26·8, 30·6]	37·2 [35·3, 39·1]	0·772 [0·758, 0·785]
250	78·6 [77·5, 79·7]	73·0 [72·6, 73·4]	21·4 [20·3, 22·5]	76·4 [75·7, 77·0]	0·842 [0·835, 0·848]	97·6 [96·8, 98·2]	18·6 [17·4, 19·9]	2·4 [1·8, 3·2]	57·5 [56·2, 58·9]	0·779 [0·767, 0·790]
251	73·5 [72·3, 74·6]	80·1 [79·8, 80·5]	26·5 [25·4, 27·7]	71·8 [71·0, 72·5]	0·845 [0·839, 0·851]	97·5 [96·8, 98·1]	19·0 [17·7, 20·3]	2·5 [1·9, 3·2]	57·4 [56·1, 58·8]	0·788 [0·776, 0·801]
270	69·4 [68·1, 70·6]	81·6 [81·3, 82·0]	30·6 [29·4, 31·9]	71·3 [70·6, 72·1]	0·829 [0·822, 0·835]	75·4 [73·6, 77·2]	65·5 [63·9, 67·0]	24·6 [22·8, 26·4]	42·6 [40·8, 44·4]	0·782 [0·770, 0·794]
271	70·5 [69·3, 71·7]	78·3 [78·0, 78·7]	29·5 [28·3, 30·7]	74·3 [73·6, 75·0]	0·824 [0·817, 0·830]	70·8 [68·9, 72·7]	74·8 [73·4, 76·2]	29·2 [27·3, 31·1]	36·6 [34·7, 38·5]	0·771 [0·758, 0·784]
289	72·1 [70·9, 73·3]	82·6 [82·2, 82·9]	27·9 [26·7, 29·1]	69·4 [68·6, 70·2]	0·850 [0·844, 0·856]	97·4 [96·6, 98·0]	18·2 [17·0, 19·5]	2·6 [2·0, 3·4]	57·7 [56·3, 59·0]	0·779 [0·767, 0·791]
311	69·1 [67·8, 70·3]	81·5 [81·1, 81·8]	30·9 [29·7, 32·2]	71·6 [70·8, 72·4]	0·831 [0·825, 0·838]	75·3 [73·5, 77·1]	65·8 [64·3, 67·4]	24·7 [22·9, 26·5]	42·4 [40·6, 44·2]	0·781 [0·769, 0·794]
380	71·3 [70·1, 72·5]	83·9 [83·6, 84·2]	28·7 [27·5, 29·9]	68·0 [67·1, 68·8]	0·851 [0·845, 0·857]	97·4 [96·6, 98·0]	18·4 [17·1, 19·7]	2·6 [2·0, 3·4]	57·6 [56·3, 59·0]	0·793 [0·782, 0·805]
392	71·4 [70·2, 72·6]	84·5 [84·2, 84·8]	28·6 [27·4, 29·8]	67·1 [66·2, 67·9]	0·854 [0·848, 0·860]	97·9 [97·2, 98·4]	16·3 [15·1, 17·5]	2·1 [1·6, 2·8]	58·1 [56·8, 59·5]	0·798 [0·787, 0·810]
402	69·6 [68·4, 70·8]	81·6 [81·3, 81·9]	30·4 [29·2, 31·6]	71·3 [70·5, 72·0]	0·833 [0·827, 0·840]	74·2 [72·4, 76·0]	68·4 [66·9, 69·9]	25·8 [24·0, 27·6]	40·9 [39·0, 42·7]	0·781 [0·769, 0·794]
417	72·0 [70·8, 73·2]	83·7 [83·4, 84·0]	28·0 [26·8, 29·2]	68·0 [67·2, 68·8]	0·856 [0·850, 0·862]	97·9 [97·2, 98·4]	16·9 [15·7, 18·2]	2·1 [1·6, 2·8]	58·0 [56·6, 59·3]	0·799 [0·787, 0·811]

Ledger: Values shown are percentages (except AUC), accompanied by 95% confidence intervals· rpart=Decision tree, XGB=eXtreme Gradient Boosting

Supplementary Table 4C: Performance characteristics by age subgroup of models shortlisted as tool candidates using the internal validation (TARN) dataset

Task ID	16-64 years					65+ years				
	Sensitivity	Specificity	Under-triage	Over-triage	AUC	Sensitivity	Specificity	Under-triage	Over-triage	AUC
RPART										
1	76·0 [74·6, 77·3]	71·8 [71·2, 72·4]	24·0 [22·7, 25·4]	66·8 [65·8, 67·8]	0·783 [0·775, 0·791]	66·3 [64·0, 68·6]	75·3 [74·8, 75·8]	33·7 [31·4, 36·0]	87·2 [86·5, 87·9]	0·738 [0·726, 0·751]
3	75·3 [73·9, 76·6]	72·4 [71·8, 73·0]	24·7 [23·4, 26·1]	66·6 [65·5, 67·5]	0·787 [0·778, 0·795]	65·7 [63·4, 67·9]	75·8 [75·4, 76·3]	34·3 [32·1, 36·6]	87·1 [86·4, 87·8]	0·741 [0·728, 0·754]
37	76·0 [74·6, 77·3]	71·8 [71·2, 72·4]	24·0 [22·7, 25·4]	66·8 [65·8, 67·8]	0·794 [0·786, 0·803]	66·3 [64·0, 68·6]	75·3 [74·8, 75·8]	33·7 [31·4, 36·0]	87·2 [86·5, 87·9]	0·746 [0·733, 0·759]
52	75·3 [73·9, 76·6]	72·4 [71·8, 73·0]	24·7 [23·4, 26·1]	66·6 [65·5, 67·5]	0·792 [0·784, 0·800]	65·7 [63·4, 67·9]	75·8 [75·4, 76·3]	34·3 [32·1, 36·6]	87·1 [86·4, 87·8]	0·745 [0·732, 0·758]
124	76·0 [74·6, 77·3]	71·8 [71·2, 72·4]	24·0 [22·7, 25·4]	66·8 [65·8, 67·8]	0·788 [0·780, 0·796]	66·3 [64·0, 68·6]	75·3 [74·8, 75·8]	33·7 [31·4, 36·0]	87·2 [86·5, 87·9]	0·742 [0·729, 0·754]
XGB										
1	76·0 [74·6, 77·3]	71·8 [71·2, 72·4]	24·0 [22·7, 25·4]	66·8 [65·8, 67·8]	0·795 [0·787, 0·804]	66·3 [64·0, 68·6]	75·3 [74·8, 75·8]	33·7 [31·4, 36·0]	87·2 [86·5, 87·9]	0·747 [0·734, 0·759]
3	75·3 [73·9, 76·6]	72·4 [71·8, 73·0]	24·7 [23·4, 26·1]	66·6 [65·5, 67·5]	0·809 [0·801, 0·817]	65·7 [63·4, 67·9]	75·8 [75·4, 76·3]	34·3 [32·1, 36·6]	87·1 [86·4, 87·8]	0·763 [0·750, 0·776]
37	82·6 [81·4, 83·8]	70·1 [69·4, 70·7]	17·4 [16·2, 18·6]	66·3 [65·3, 67·2]	0·839 [0·831, 0·847]	67·3 [65·0, 69·5]	75·1 [74·6, 75·6]	32·7 [30·5, 35·0]	87·1 [86·4, 87·8]	0·752 [0·739, 0·765]
38	76·8 [75·4, 78·1]	71·5 [70·9, 72·2]	23·2 [21·9, 24·6]	66·8 [65·8, 67·7]	0·811 [0·803, 0·819]	67·2 [64·9, 69·4]	74·9 [74·4, 75·4]	32·8 [30·6, 35·1]	87·3 [86·6, 88·0]	0·765 [0·752, 0·778]
41	76·0 [74·6, 77·3]	71·8 [71·2, 72·4]	24·0 [22·7, 25·4]	66·8 [65·8, 67·8]	0·821 [0·813, 0·829]	66·3 [64·0, 68·6]	75·3 [74·8, 75·8]	33·7 [31·4, 36·0]	87·2 [86·5, 87·9]	0·766 [0·752, 0·779]
42	76·0 [74·6, 77·3]	71·8 [71·2, 72·4]	24·0 [22·7, 25·4]	66·8 [65·8, 67·8]	0·806 [0·798, 0·815]	66·3 [64·0, 68·6]	75·3 [74·8, 75·8]	33·7 [31·4, 36·0]	87·2 [86·5, 87·9]	0·778 [0·766, 0·791]
43	74·4 [73·0, 75·7]	74·0 [73·4, 74·6]	25·6 [24·3, 27·0]	65·4 [64·4, 66·4]	0·815 [0·807, 0·824]	60·0 [57·7, 62·4]	81·5 [81·1, 81·9]	40·0 [37·6, 42·3]	85·0 [84·1, 85·8]	0·760 [0·746, 0·774]
44	82·0 [80·8, 83·2]	70·6 [70·0, 71·2]	18·0 [16·8, 19·2]	66·0 [65·0, 67·0]	0·852 [0·845, 0·859]	66·7 [64·4, 68·9]	75·7 [75·2, 76·1]	33·3 [31·1, 35·6]	87·0 [86·3, 87·7]	0·769 [0·756, 0·781]
53	75·3 [73·9, 76·6]	72·4 [71·8, 73·0]	24·7 [23·4, 26·1]	66·6 [65·5, 67·5]	0·813 [0·805, 0·821]	65·7 [63·4, 67·9]	75·8 [75·4, 76·3]	34·3 [32·1, 36·6]	87·1 [86·4, 87·8]	0·780 [0·767, 0·792]
54	74·9 [73·5, 76·3]	73·6 [73·0, 74·2]	25·1 [23·7, 26·5]	65·6 [64·6, 66·7]	0·825 [0·817, 0·833]	61·2 [58·9, 63·5]	80·5 [80·1, 80·9]	38·8 [36·5, 41·1]	85·4 [84·5, 86·2]	0·773 [0·760, 0·786]
121	83·4 [82·2, 84·5]	69·8 [69·2, 70·4]	16·6 [15·5, 17·8]	66·2 [65·3, 67·2]	0·855 [0·848, 0·862]	68·1 [65·9, 70·3]	74·7 [74·2, 75·2]	31·9 [29·7, 34·1]	87·2 [86·5, 87·9]	0·770 [0·758, 0·783]
124	77·5 [76·1, 78·8]	78·8 [78·2, 79·3]	22·5 [21·2, 23·9]	59·7 [58·6, 60·8]	0·854 [0·847, 0·862]	59·1 [56·7, 61·4]	84·0 [83·6, 84·4]	40·9 [38·6, 43·3]	83·3 [82·3, 84·2]	0·772 [0·758, 0·785]
125	82·3 [81·1, 83·5]	70·5 [69·9, 71·2]	17·7 [16·5, 18·9]	66·0 [65·0, 66·9]	0·854 [0·846, 0·861]	67·2 [64·9, 69·4]	75·7 [75·2, 76·1]	32·8 [30·6, 35·1]	86·9 [86·2, 87·6]	0·784 [0·772, 0·797]
130	76·6 [75·3, 77·9]	71·6 [71·0, 72·2]	23·4 [22·1, 24·7]	66·7 [65·7, 67·7]	0·814 [0·806, 0·822]	67·3 [65·0, 69·5]	74·9 [74·4, 75·4]	32·7 [30·5, 35·0]	87·2 [86·5, 87·9]	0·783 [0·771, 0·795]
131	75·6 [74·2, 76·9]	73·7 [73·1, 74·3]	24·4 [23·1, 25·8]	65·4 [64·3, 66·4]	0·828 [0·820, 0·836]	61·7 [59·4, 64·0]	80·4 [80·0, 80·8]	38·3 [36·0, 40·6]	85·3 [84·5, 86·1]	0·775 [0·761, 0·788]
141	74·8 [73·4, 76·1]	73·7 [73·1, 74·3]	25·2 [23·9, 26·6]	65·6 [64·6, 66·6]	0·824 [0·816, 0·833]	62·4 [60·0, 64·7]	80·6 [80·2, 81·1]	37·6 [35·3, 40·0]	85·1 [84·2, 85·9]	0·788 [0·775, 0·800]
154	75·5 [74·1, 76·9]	77·1 [76·5, 77·7]	24·5 [23·1, 25·9]	62·2 [61·1, 63·3]	0·847 [0·839, 0·854]	61·8 [59·4, 64·1]	78·6 [78·2, 79·1]	38·2 [35·9, 40·6]	86·4 [85·6, 87·1]	0·780 [0·768, 0·793]
165	72·9 [71·5, 74·3]	78·3 [77·8, 78·9]	27·1 [25·7, 28·5]	61·7 [60·6, 62·8]	0·838 [0·830, 0·846]	59·2 [56·8, 61·5]	83·9 [83·5, 84·3]	40·8 [38·5, 43·2]	83·3 [82·4, 84·2]	0·781 [0·768, 0·794]
166	75·9 [74·5, 77·2]	72·2 [71·5, 72·8]	24·1 [22·8, 25·5]	66·5 [65·5, 67·5]	0·828 [0·820, 0·836]	62·8 [60·5, 65·1]	78·9 [78·5, 79·4]	37·2 [34·9, 39·5]	86·0 [85·2, 86·8]	0·790 [0·778, 0·803]
250	83·2 [82·0, 84·3]	70·1 [69·4, 70·7]	16·8 [15·7, 18·0]	66·1 [65·1, 67·1]	0·860 [0·853, 0·868]	68·3 [66·0, 70·4]	74·9 [74·4, 75·4]	31·7 [29·6, 34·0]	87·1 [86·4, 87·8]	0·789 [0·777, 0·801]
251	79·9 [78·6, 81·2]	75·2 [74·6, 75·8]	20·1 [18·8, 21·4]	62·7 [61·7, 63·8]	0·863 [0·856, 0·871]	58·9 [56·6, 61·3]	83·4 [83·0, 83·8]	41·1 [38·7, 43·4]	83·8 [82·8, 84·7]	0·783 [0·770, 0·796]
270	73·7 [72·3, 75·1]	78·2 [77·6, 78·7]	26·3 [24·9, 27·7]	61·6 [60·5, 62·7]	0·840 [0·833, 0·848]	59·6 [57·2, 61·9]	83·9 [83·5, 84·3]	40·4 [38·1, 42·8]	83·2 [82·2, 84·1]	0·783 [0·770, 0·796]
271	74·5 [73·1, 75·9]	74·6 [74·0, 75·2]	25·5 [24·1, 26·9]	64·9 [63·8, 65·9]	0·830 [0·822, 0·838]	61·6 [59·2, 63·9]	80·8 [80·3, 81·2]	38·4 [36·1, 40·8]	85·1 [84·3, 85·9]	0·791 [0·779, 0·804]
289	77·5 [76·1, 78·8]	80·2 [79·7, 80·7]	22·5 [21·2, 23·9]	58·1 [56·9, 59·2]	0·869 [0·862, 0·876]	60·2 [57·8, 62·5]	84·1 [83·7, 84·5]	39·8 [37·5, 42·2]	82·9 [81·9, 83·8]	0·797 [0·785, 0·810]
311	73·1 [71·6, 74·5]	78·1 [77·5, 78·6]	26·9 [25·5, 28·4]	61·9 [60·8, 63·0]	0·840 [0·832, 0·847]	60·1 [57·7, 62·4]	83·7 [83·3, 84·1]	39·9 [37·6, 42·3]	83·3 [82·3, 84·2]	0·797 [0·784, 0·809]
380	76·9 [75·5, 78·2]	81·6 [81·0, 82·1]	23·1 [21·8, 24·5]	56·5 [55·3, 57·7]	0·870 [0·863, 0·877]	58·7 [56·3, 61·0]	85·4 [85·1, 85·8]	41·3 [39·0, 43·7]	82·0 [80·9, 83·0]	0·798 [0·786, 0·810]
392	77·2 [75·9, 78·5]	80·8 [80·3, 81·3]	22·8 [21·5, 24·1]	57·4 [56·2, 58·6]	0·869 [0·862, 0·876]	58·3 [55·9, 60·6]	87·0 [86·6, 87·4]	41·7 [39·4, 44·1]	80·3 [79·2, 81·4]	0·801 [0·788, 0·813]
402	73·6 [72·2, 75·0]	78·2 [77·6, 78·7]	26·4 [25·0, 27·8]	61·6 [60·5, 62·8]	0·842 [0·834, 0·849]	60·6 [58·2, 62·9]	83·9 [83·5, 84·3]	39·4 [37·1, 41·8]	83·0 [82·0, 83·9]	0·797 [0·785, 0·810]
417	77·9 [76·5, 79·2]	80·2 [79·7, 80·8]	22·1 [20·8, 23·5]	57·9 [56·7, 59·0]	0·873 [0·866, 0·880]	58·8 [56·4, 61·1]	86·0 [85·6, 86·4]	41·2 [38·9, 43·6]	81·4 [80·3, 82·4]	0·803 [0·790, 0·815]

Ledger: Values shown are percentages (except AUC), accompanied by 95% confidence intervals· rpart=Decision tree, XGB=eXtreme Gradient Boosting
